# Characterization of Three Novel SXT/R391 Integrating Conjugative Elements ICE*Mfu*Ind1a and ICE*Mfu*Ind1b, and ICE*Mpr*Chn1 Identified in the Genomes of *Marinomonas fungiae* JCM 18476^T^ and *Marinomonas profundimaris* Strain D104

**DOI:** 10.3389/fmicb.2016.01896

**Published:** 2016-11-25

**Authors:** Jhasketan Badhai, Subrata K. Das

**Affiliations:** Department of Biotechnology, Institute of Life SciencesBhubaneswar, India

**Keywords:** SXT/R391 ICEs, mobile genetic elements, *Marinomonas*, genomic analysis, hotspots

## Abstract

The genus *Marinomonas* comprises Gram negative bacteria which are widespread in the marine environment and there is no report on the genomic analysis of SXT/R391 ICEs derived from this group of bacteria. This study describes the genomic features of three new SXT/R391 integrating conjugating elements (ICEs) identified in the genome of *Marinomonas fungiae* JCM 18476^T^ (ICE*Mfu*Ind1a and ICE*Mfu*Ind1b) and in *Marinomonas profundimaris* strain D104 (ICE*Mpr*Chn1). Structural organizations of the three ICEs were similar to the typical SXT/R391 family of ICEs and showed high degree of conservation in the core genes. Sequence analysis revealed ICE*Mfu*Ind1b and ICE*Mpr*Chn1 were inserted into the genome at 5′-end of an typical host *prfC* gene, while ICE*Mfu*Ind1a was inserted at 5′-end of an atypical *hipA*-like gene. Despite their coexistence, the ICE*Mfu*Ind1a and ICE*Mfu*Ind1b were not present in a tandem fashion in the genome of *M. fungiae.* Phylogenetic analyses revealed the three ICEs either evolved independently or high degrees of recombination events had masked their evolution from a common SXT ancestor. Further, we found that the typical entry exclusion mechanism mediated by the TraG/EeX protein pair was likely defective in preventing the conjugative transfer of a second copy of the same S (SXT) group ICE into the *M. fungiae* genome due to mutations. Our analysis showed the presence of 16, 25, and 27 variable genes in the hotspots of ICE*Mfu*Ind1a, ICE*Mfu*Ind1b, and ICE*Mpr*Chn1, respectively, many of which were not reported earlier for SXT/R391 ICEs. Sequence analysis predicted these hotspot regions were shaped by acquisition of genes through homologous recombination between the SXT and R391 related ICEs or mobile genetic elements present in disparate marine bacteria. Multidrug resistance genes which are hallmark feature of SXT/R391 ICEs were not present in either of the two ICEs from *M. fungiae* but were present within a transposon cassette in the HS-1 of the ICE*Mpr*Chn1 from *M. profundimaris.* Finally, our data provided information on the genetic diversity and predicted functions encoded by variable genes present in the hotspot regions of these new ICEs.

## Introduction

Integrating conjugative elements (ICEs) are self-transmissible mobile genetic elements (MGEs) that are widely distributed in bacterial genomes and play a major role in bacterial adaptation, genome dynamics, and evolution (Beaber et al., 2004; Burrus and Waldor, 2004a; Bi et al., 2012; Carraro and Burrus, 2014; Johnson and Grossman, 2015). The ICEs of the SXT/R391 family are major drivers in the dissemination of heavy metals and multidrug resistance among environmental and pathogenic clinical strains of diverse bacterial groups within the *Gammaproteobacteria* (Burrus et al., 2006; Wozniak et al., 2009; Bi et al., 2012; Johnson and Grossman, 2015). To date, SXT/R391 ICEs have been found in several species of *Vibrio, Shewanella, Photobacterium, Providencia*, and *Proteus* (Hochhut et al., 2001; Ahmed et al., 2005; Pembroke and Piterina, 2006; Osorio et al., 2008; Wozniak et al., 2009; Harada et al., 2010; Rodríguez-Blanco et al., 2012; Spagnoletti et al., 2014). The prototypical elements of this family of ICEs i.e., SXT and R391 were derived from *Vibrio cholerae* O139 in India and *Providencia rettgeri* in South Africa, respectively (Coetzee et al., 1972; Waldor et al., 1996). All the SXT/R391 ICEs are chromosomal MGEs sharing a conserved integrase that mediates site-specific integration into the 5′ end of *prfC* or *t-RNA-ser* in the absence of a *prfC* site (Hochhut and Waldor, 1999; Hochhut et al., 2001; Burrus and Waldor, 2003; Burrus et al., 2006; Taviani et al., 2012; Carraro and Burrus, 2014; Luo et al., 2016). Members of this ICE family contain 52 conserved core genes, many of which are involved in integration/excision, conjugative transfer and regulation of the ICEs (Beaber et al., 2002; Burrus et al., 2006; Wozniak et al., 2009; Bi et al., 2012; Spagnoletti et al., 2014; Carraro et al., 2015; Poulin-Laprade and Burrus, 2015; Poulin-Laprade et al., 2015). In addition, five hotspots (HS1—5) and five variable (VRI—V) regions have also been identified (Lei et al., 2016), which contain variable genes conferring element-specific properties and providing beneficial phenotypes to their hosts (Osorio et al., 2008; Wozniak et al., 2009; Rodríguez-Blanco et al., 2012; Balado et al., 2013; Poulin-Laprade et al., 2015). It has been demonstrated that genes encoding for resistance to multiple antibiotics and heavy metals, aromatic compound degradation pathways, DNA repair and recombination systems, virulence factors, toxin-antitoxin system, regulation of motility, and biofilm formation are found to be present within the hotspots and variable regions in the ICEs of many bacteria (Boltner et al., 2002; Wozniak et al., 2009; Rodríguez-Blanco et al., 2012; Balado et al., 2013). However, information on the dissemination and ecology of ICEs in marine environment is limited. Apart from SXT/R391, the other families of ICEs that are present widespread in Gram negative and Gram positive bacteria and studied extensively to understand their biology and evolution are ICE*Bs1* from *Bacillus subtilis*, ICE*St1*/ICE*St3* from *Streptococcus thermophilus*, ICE*clc* from *Pseudomonas putida*, ICE*Hin1056* from *Haemophilus influenza*, ICE*Lm1* from *Listeria monocytogenes*, etc. (Carraro and Burrus, 2014; Johnson and Grossman, 2015).

Previously, laboratory experiments with *E. coli* and *V. cholerae* have demonstrated the transfer of SXT and R391 ICEs often results in the formation of exconjugants harboring multiple copies of SXT integrated within the 5′ end of *prfC* gene in tandem arrays (Hochhut et al., 2001; Burrus and Waldor, 2004b). Further, Marrero and Waldor (2005, 2007) in their studies have shown that the SXT/R391 family of ICEs is divided into two exclusion groups: the S (SXT) and R (R391). It has been demonstrated that cells containing SXT, exclude transfer of a second copy of SXT but not R391 and vice versa which is mediated by variants of the two cognate inner membrane proteins, TraG and Eex, in donor and recipient cells, respectively. Moreover, these ICE tandem arrays do not persist in the *recA*^+^ strains and are quickly brought down to a singleton state after a few generations by homologous recombination mediated by host RecA and ICE Bet/Exo (Garriss et al., 2009, 2013).

The coexistence of two ICEs of the same exclusion group either S (SXT) or R (R391) in a genome is very rare (Marrero and Waldor, 2007) and limited information is available on natural isolates harboring such SXT/R391 ICEs arrays (Luo et al., 2016). Moreover, there is no report on the genomic analysis of SXT/R391 ICEs derived from the members of the genus *Marinomonas*. Thus, in the present study we described and compared the genetic features of three new SXT ICEs. Among them two were identified in the genome of *M. fungiae* JCM 18476^T^ and one in the previously sequenced genome of *M. profundimaris* strain D104 (Dong et al., 2014).

## Materials and methods

### Bacterial strain and media

*Marinomonas fungiae* JCM 18476^T^ positive for SXT/R391 family related ICEs was used for genomic analysis (Badhai et al., 2013). The bacterium was routinely grown in marine agar 2216 (MA; Difco) at 28°C (Kumari et al., 2014).

### Genomic DNA preparation, sequencing, and assembly

The genomic DNA of *Marinomonas fungiae* JCM 18476^T^ was isolated using standard methods (Sambrook et al., 1989). The draft genome of *Marinomonas fungiae* JCM 18476^T^ was generated at the DOE Joint Genome Institute (JGI, USA) using the Illumina HiSeq 2000 platform (Bennett, 2004). The genome was annotated using the JGI Microbial Genome Annotation Pipeline (Mavromatis et al., 2009). The methods for the genomic DNA preparation, sequencing, assembly and annotation of *M. profundimaris* strain D104 was described by Dong et al. (2014).

### Comparative analysis of ICEs

The genetic organizations of the three ICEs derived from *M. fungiae* JCM 18476^T^ (ICE*Mfu*Ind1a and ICE*Mfu*Ind1b) and *M. profundimaris* strain D104 (ICE*Mpr*Chn1) were determined by comparison with the core backbone structures of 11 reference SXT/R391 ICEs from *Providencia rettgeri, Vibrio cholerae* O139 and O1 strains, *Shewanella putrefaciens, Vibrio fluvialis, Photobacterium damselae, Providencia alcalifaciens*, and *Proteus mirabilis* (Wozniak et al., 2009; Lei et al., 2016). We considered only 11 reference SXT/R391 ICEs for comparative analysis as their complete genomic information were available and well characterized from the ICEberg database as on 24th August, 2011 and NCBI-RefSeq database as on 29th May, 2016. Sequence conservation at nucleotide and amino acid levels, as well as presence or absence of genes/ORFs with respect to reference SXT/R391 ICEs was determined using BLAST (Altschul et al., 1997) locally (standalone BLAST−2.2.29+ package; Camacho et al., 2009). In addition, identification of the genes/ORFs present in the hotspot regions was carried out using BLASTX against the NCBI-RefSeq and ICEberg (http://db-mml.sjtu.edu.cn/ICEberg/; Bi et al., 2012) databases. Clustal omega was used for sequence alignments (the program is available at http://www.ebi.ac.uk/Tools/msa/clustalo/). DNAPlotter was used to generate images of the linear DNA maps (Carver et al., 2009).

### Analysis of the excision abilities of the ICEs

Polymerase chain reaction (PCR) was performed targeting the reconstituted *attP* sites of the circular extra chromosomal form of the ICEs using forward 5′-TGCTGTCATCTGCATTCTCCTG-3′ and reverse 5′-GCCAATTACGATTAACACGACGG-3′ primers (Hochhut and Waldor, 1999) to verify the excision abilities of the two ICEs of *M. fungiae* JCM 18476^T^.

### Phylogenetic analysis of core ICE genes

Phylogenetic analysis was performed based on the concatenated amino acid sequences of 25 core genes encoded proteins: Int, SrpR, SrpM, RumA, S024, TraE, TraK, TraV, TraA, S054, TraC, TrhF, TraU, TraN, S063, Ssb, Bet, Exo, TraF, TraH, TraG, EeX, SetC, SetD, and SetR. In addition, individual phylogenetic analysis was performed for the proteins: Int, TraI, TraG, Eex, Bet, and Exo. Phylogenetic trees were constructed by maximum-likelihood method based on the Poisson correction model (Zuckerkandl and Pauling, 1965) using the MEGA6 (Tamura et al., 2013). Bootstrap analysis with 1000 replications was performed to test the reliability of the tree. Reference ICEs sequences were retrieved from GenBank: SXT^MO10^ (*V. cholerae* O139; accession: AY055428), ICE*Vch*Ind4 (*V. cholera* O139; accession: GQ463141), ICE*Vch*Ind5 (*V. cholera* O1; accession: GQ463142), ICE*Vch*Ban5 (*V. cholera* O1; accession: GQ463140), ICE*Vch*Mex1 (*V. cholerae* non O139; accession: GQ463143), R391 (*Providencia rettgeri*; accession: AY090559), ICE*Pal*Ban1 (*Providencia alcalifaciens*; accession: GQ463139), ICE*Vfl*Ind1 (*V. fluvialis*; accession: GQ463144), ICE*Pda*Spa1 (*Photobacterium damselae*; accession: AJ870986), ICE*Spu*PO1 (*Shewanella putrefaciens*; accession: CP000503), and ICE*Pmi*Chn1 (*Proteus mirabilis*; accession: KT962845).

### Nucleotide sequence accession number

The draft genome sequence of *M. fungiae* JCM 18476^T^ and *M. profundimaris* strain D104 (Dong et al., 2014) are available at NCBI GenBank under the accession no.'s: LIQF00000000 and AYOZ00000000, respectively. The versions described in this paper are version LIQF01000000 and AYOZ01000000, respectively.

## Results and discussion

### Assembly of the ICEs

Genomic analysis revealed co-existence of two ICEs in the genome of *M. fungiae* and one ICE in the genome of *M. profundimaris*. The three ICEs were assembled based on sequence similarity and structural comparison with the backbone of core genes in SXT/R391 ICEs. The two ICEs of *M. fungiae* were designated as ICE*Mfu*Ind1a and ICE*Mfu*Ind1b, and the *M. profundimaris* was designated as ICE*Mpr*Chn1. The ICE*Mfu*Ind1a and ICE*Mfu*Ind1b were encoded by three (GenBank accession: LIQF01000019.1, LIQF01000033.1, and LIQF01000014.1) and four (GenBank accession: LIQF01000022.1, LIQF01000023.1, LIQF01000030.1, and LIQF01000009.1) DNA scaffolds of total length 65.5 and 74.7 kb, respectively, whereas the ICE*Mpr*Chn1 was encoded by four (GenBank accession: AYOZ01000034.1, AYOZ01000017.1, AYOZ01000022.1, and AYOZ01000004.1) DNA scaffolds of total length 86.4 kb. BLAST search showed a homolog of the chromosomal *prfC* gene was present adjacent to the *attR* sequence, consistent with the SXT/R391 ICE insertion site (Hochhut and Waldor, 1999; Hochhut et al., 2001; Burrus and Waldor, 2003) at the extreme 3′-end of the ICE*Mfu*Ind1b and ICE*Mpr*Chn1, but it was absent in ICE*Mfu*Ind1a. Instead, the ICE*Mfu*Ind1a was inserted at the 5′-end of a putative *hipA*-like toxin gene. Further, the two ICEs of *M. fungiae* were not arranged in a tandem fashion in the genome, there were several non-ICE genes that immediately followed the predicted *setR* and the *setR*-*prfC* locus at the extreme 3′-end of ICE*Mfu*Ind1a and ICE*Mfu*Ind1b, respectively.

### Structural organization and general features of the core ICE backbones

**Figure 1:**
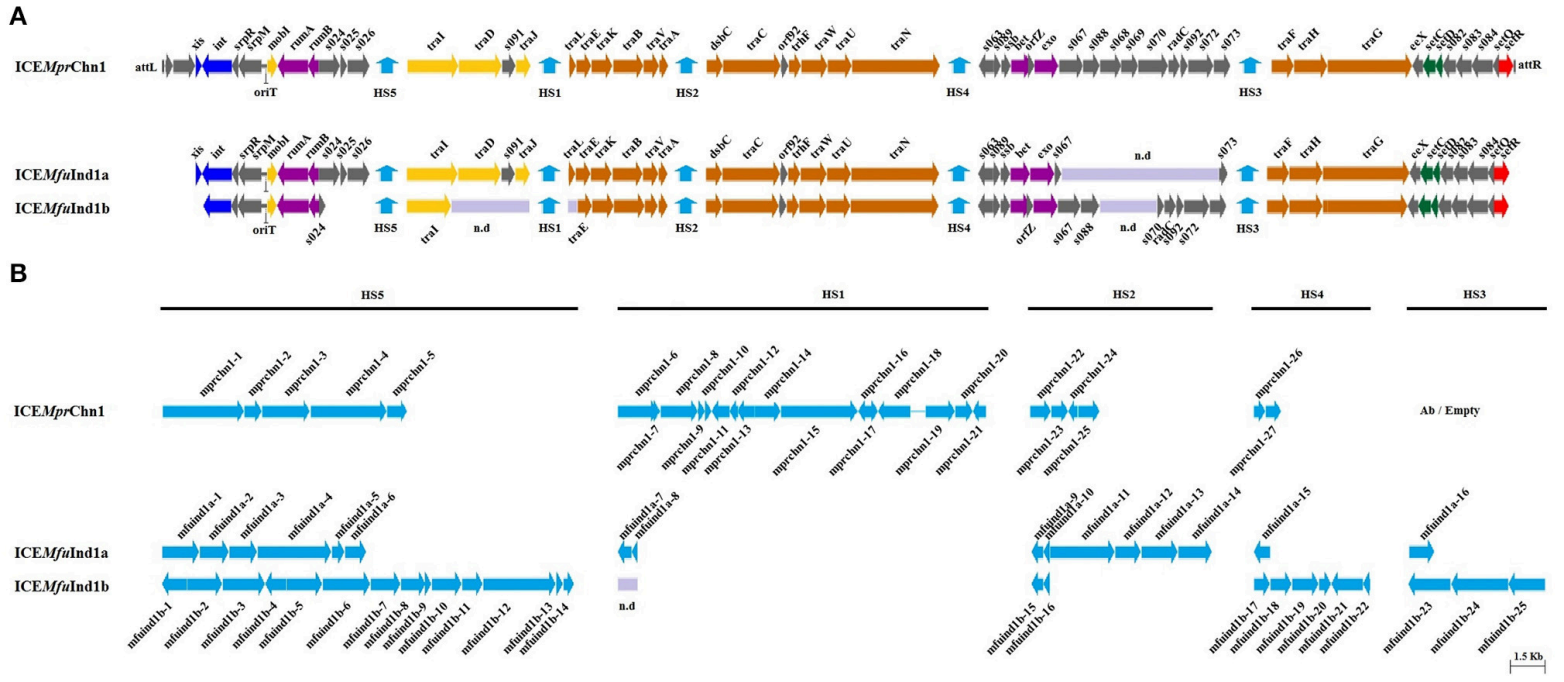
**Schematic representation of the (A)**, core SXT-like ICE backbones and **(B)**, the five hotspot regions of the three ICEs: ICE*Mfu*Ind1a, ICE*Mfu*Ind1b, and ICE*Mpr*Chn1. Genes/ORFs in the five hotspots are designated as a1–a16 and b1–b25 in ICE*Mfu*Ind1a and ICE*Mfu*Ind1b, respectively (see **Table 2**). ICE*Mfu*Ind1a was encoded by the scaffolds: LIQF01000019.1, LIQF01000033.1, and LIQF01000014.1; ICE*Mfu*Ind1b by LIQF01000022.1, LIQF01000023.1, LIQF01000030.1, and LIQF01000009.1; and ICEMprChn1 by: AYOZ01000034.1, AYOZ01000017.1, AYOZ01000022.1, and AYOZ01000004.1. Genes/ORFs are color coded: blue, integration, and excision; yellow, DNA processing; orange, conjugative transfer system; purple, RecA-independent homologous recombination, and Umu-like mutagenic repair; green, transcriptional activator; red, transcriptional repressor; gray, other or hypothetical functions; cyan, hotspot genes; violet, gap regions; n.d, sequence not determined.

The three elements were not identical; they exhibited variability in the degree of sequence conservation when compared with other SXT/R391 ICEs. Moreover, G+C content was 44.8, 47.2, and 46.9% for ICE*Mfu*Ind1a, ICE*Mfu*Ind1b, and ICE*Mpr*Chn1, respectively. Most of the core genes of SXT/R391 ICEs (Beaber et al., 2002; Wozniak et al., 2009; Spagnoletti et al., 2014; Carraro et al., 2015; Poulin-Laprade and Burrus, 2015; Poulin-Laprade et al., 2015) were found to be preserved and were arranged in the same syntenic order in the three elements (Figure 1A, Supplementary Table 1) and showed 60–99% sequence identity at the level of amino acid to the corresponding proteins encoded by SXT/R391 ICEs (Supplementary Table 2).

Among the conserved core genes, the homologs of genes encoding the excision and integration functions (*xis* and *int* at the extreme 5′ end), plasmid-like partition system (*srpRMC*), a Umu-like mutagenic DNA repair system (*rumAB*), a RecA-independent λ Red-like homologous recombination system (*bet*/*exo*), five conjugative DNA processing and transfer clusters (*mobI, traIDJ, traLEKBVA, dsbC*/*traC*/*trhF*/*traWUN*, and *traFHG*), regulators of the excision and conjugative transfer (*setCD, croS*, and *setR*) were present in all the three ICEs analyzed (Figure 1A, Supplementary Table 1). However, the sequences of the genes *xis, traD, s091, traJ*, and *traL* were not determined in the case of ICE*Mfu*Ind1b. Further, PCR assay showed the amplification of a 785 bp DNA fragment containing the reconstituted *attP* sites of the circular extrachromosomal form of the ICE (Hochhut and Waldor, 1999) suggesting the ICEs are excised from the genome of *M. fungiae* JCM 18476^T^ (data not shown). In addition, sequence analysis showed all the three ICEs carried an intact *rumB* gene; lacked the antibiotic resistance gene clusters inserted into the *rumB* gene, a characteristics typical to many ICEs belonging to the SXT/R391 family. Analysis of the intergenic region between *srpM* and *mobI* in all the three ICEs showed a high degree of sequence conservation with the other eleven ICEs used for comparison (Supplementary Figure 1). This region presumably encoded the 299 bp long putative origin of transfer (*oriT*), where the conjugative DNA transfer is typically initiated following the excision of the ICE from the chromosome (Ceccarelli et al., 2008).

We found low identity of the predicted amino acid sequence of the 3′ regulatory module (consisting of the eight genes *setCD, s082, s083, s084, croS*, and *setR*) in ICE*Mfu*Ind1a in comparison with those encoded by the conserved core genes of other SXT/R391 ICEs. Therefore, it could be predicted that this newly identified ICE was generated as a result of homologous recombination between two different SXT/R391 ICEs as demonstrated by Garriss et al. (2009, 2013).

Structural variations were also observed in the three ICEs, including in the core backbones and in the five variable DNA regions, termed hotspots (Wozniak et al., 2009; Figure 1). The highly conserved *orfZ* gene found between *bet* and *exo* in SXT/R391 ICEs was absent in ICE*Mfu*Ind1a, whereas there was disruption in the 3′-end of the gene *s024* and deletion of the entire genes *s025* and *s026* in ICE*Mfu*Ind1b. However, functions of these genes in the conjugative ICE transfer are unknown (Beaber et al., 2002; Wozniak et al., 2009). In ICE*Mpr*Chn1, the typical hotspot-3 was empty/without variable DNA.

### Exclusion system

**Figure 2:**
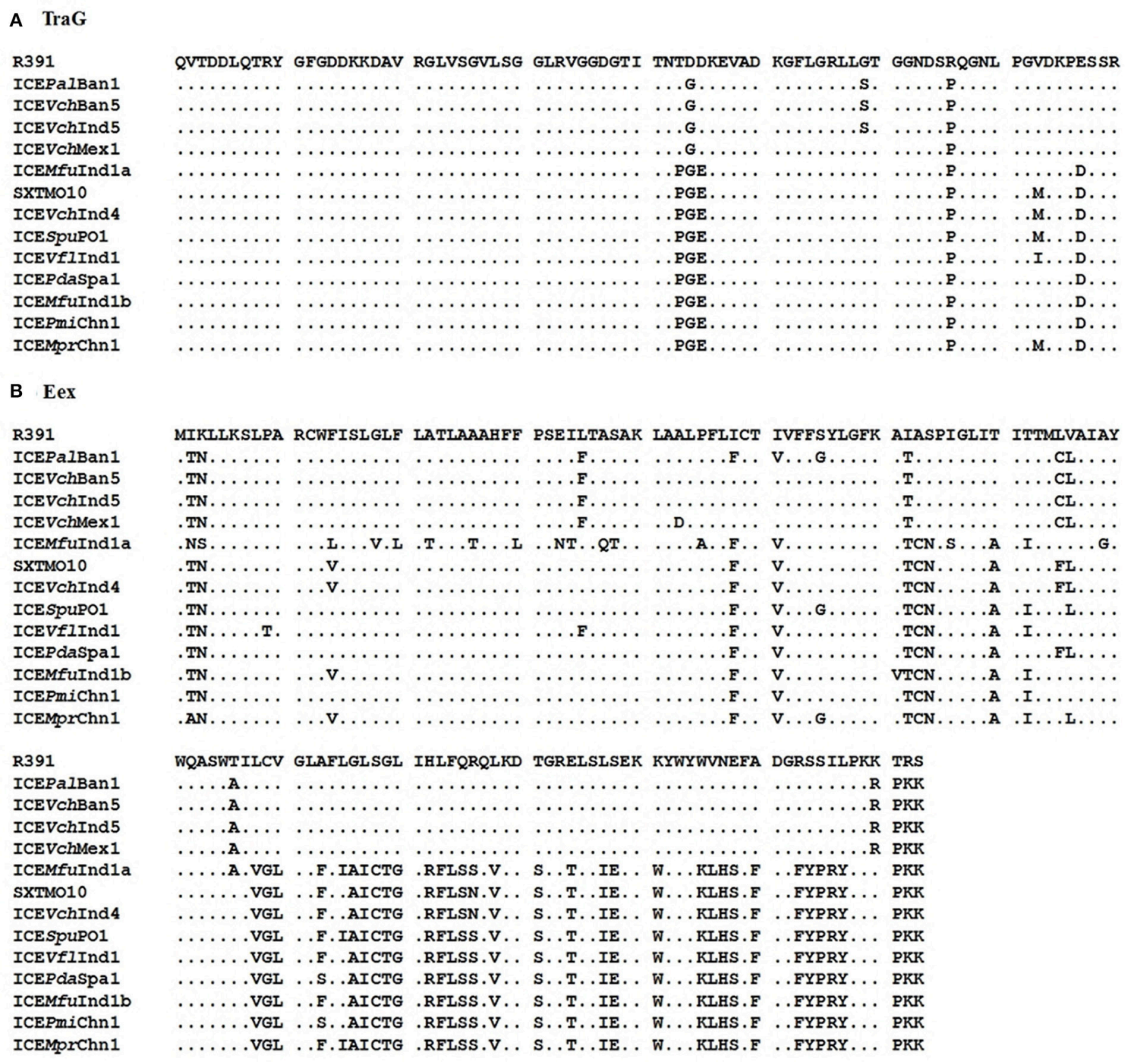
**Clustal Omega alignment of the (A)**, TraG and **(B)**, Eex protein sequences obtained from the three ICEs: ICE*Mfu*Ind1a, ICE*Mfu*Ind1b, and ICE*Mpr*Chn1, with the corresponding sequences from the 11 reference SXT/R391 ICEs retrieved from the GenBank, showing the predicted SXT-like exclusion amino acids in TraG (P-G-E) and Eex proteins.

Marrero and Waldor (2005, 2007) have shown that the ICE entry exclusion specificity is determined by the carboxyl terminal residues in the Eex exclusion proteins, but the exclusion potency varied to a large extent on the basis of change in a single amino acid (i.e., 43rd residue) at the amino terminal of this protein. In the present study, all the three ICEs were found to be from the S entry exclusion group. Amino acid sequence alignment of the TraG proteins showed that the predicted S exclusion determinant residues were P-G-E in all the three ICEs (Figure 2A). Similarly, the alignment of Eex exclusion proteins predicted that the three ICEs encoded proteins that belonged to the S exclusion group (Figure 2B). However, we found variations in 16 amino acid residues at the amino terminal region of Eex exclusion protein encoded by ICE*Mfu*Ind1a which may have reduced the exclusion potency to a very large extent, thereby allowing the acquisition of a second copy of the SXT-like ICE (ICE*Mfu*Ind1b) into the *M. fungiae* genome.

### Phylogenetic analysis of the core ICE genes

**Figure 3:**
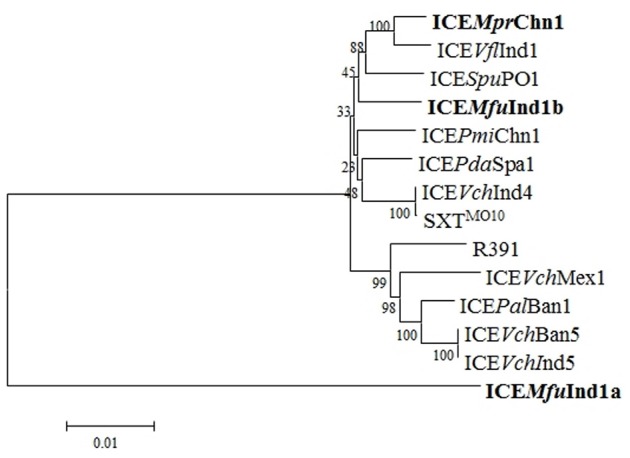
**Phylogenetic analysis of core ICE genes encoded proteins**. The tree was constructed by applying the Maximum Likelihood method based on the Poisson correction model using the MEGA6. Bootstrap analysis with 1000 replications was performed to test the reliability of the tree.

**Figure 4:**
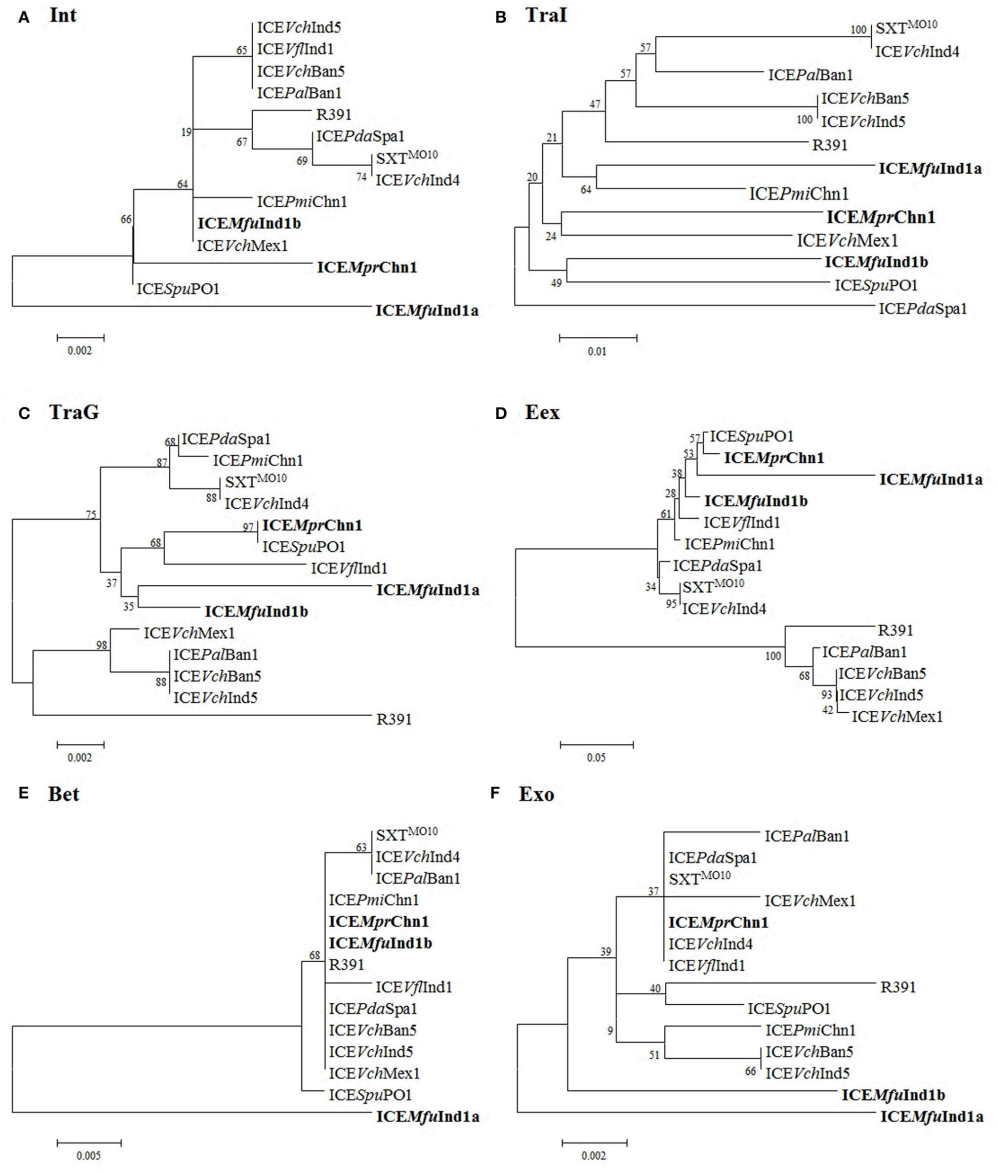
**Phylogenetic analysis of (A)** Int, **(B)** TraI, **(C)** TraG, **(D)** Eex, **(E)** Bet, and **(F)** Exo protein. The trees were constructed by applying the Maximum Likelihood method based on the Poisson correction model using MEGA6. Bootstrap analysis with 1000 replications was performed to test the reliability of each tree.

A phylogenetic tree was constructed based on the concatenated amino acid sequences of 25 core proteins: Int, SrpR, SrpM, RumA, S024, TraE, TraK, TraV, TraA, S054, TraC, TrhF, TraU, TraN, S063, Ssb, Bet, Exo, TraF, TraH, TraG, EeX, SetC, SetD, and SetR encoded by ICE*Mfu*Ind1a and ICE*Mfu*Ind1b derived from *M. fungiae* JCM 18476^T^, ICE*Mpr*Chn1 from *M. profundimaris* strain D104 and the other 11 reference SXT/R391 ICEs to trace the evolution of these ICEs. Our analysis showed the three ICEs were clustering into two branches; while ICE*Mfu*Ind1b and ICE*Mpr*Chn1 were very closely related and clustered with the SXT ICEs, the ICE*Mfu*Ind1a was distantly related to either SXT or R391 ICEs and formed a separate branch (Figure 3, Supplementary Figure 2). To further explore the evolutionary relationship between the ICEs and infer their ancestral root, we created phylogenetic trees for six proteins (Figure 4) involved in most important ICE functions, such as integration (Int), transfer (TraI), exclusion determination (TraG and Eex), and recombination (Bet and Exo). The different clustering of branches in the phylogenetic trees corroborated that most likely these ICEs have evolved independently or high degrees of recombination events have masked their evolution from a common SXT ancestor (Garriss et al., 2009, 2013; Wozniak et al., 2009).

### Genetic analysis of the hotspot regions

Despite showing similarity with the conserve core backbones, all the three ICEs derived from the two bacteria of the genus *Marinomonas* carried variable genes clustered in the five conserved insertion hotspots (Wozniak et al., 2009; Figure 1). The boundaries of the five hotspots were located between *s026* and *traI* (HS-5), *traJ*, and *traL* (HS-1, sequence not determined in case of ICE*Mfu*Ind1b), *traA* and *s054* (HS-2), *s073* and *traF* (HS-4), and *traN* and *s063* (HS-3) in both the ICEs, except the HS-5 in ICE*Mfu*Ind1b (Tables 1–**3**). The left boundary of the HS-5 of ICE*Mfu*Ind1b was within *s024* instead of being located downstream to *s026* at the 3′ end. These hotspots varied in size from 902 to 9784, 1173 to 19,100, and 1348 to 15,615 bp in ICE*Mfu*Ind1a, in ICE*Mfu*Ind1a, and ICE*Mpr*Chn1, respectively.

**Table 1:** **Descriptions of genes or ORFs present in the hotspot regions of ICE*Mfu*Ind1a**.

**Hotspot region**	**ICE*Mfu*Ind1a gene/ORF id**	**Gene name**	**Length (bp)**	**Hotspot gene product name**	**GenBank accession no. of protein homolog**	**% Similarity with homolog**
HS-5 (*s026*-*traI*)	Ga0061065_11938	mfuind1a-1	1560	Type-I restriction enzyme M subunit	*Psychromonas arctica*; WP_028870232.1	99
	Ga0061065_11937	mfuind1a-2	1245	Type-I restriction enzyme S subunit	*Psychromonas arctica*; WP_028870233.1	70
	Ga0061065_11936	mfuind1a-3	1176	Hypothetical protein	*Photobacterium aquae*; WP_047879742.1	95
	Ga0061065_11935	mfuind1a-4	3117	Type-I restriction enzyme R subunit	*Psychromonas arctica*; WP_028870235.1	99
	Ga0061065_11934	mfuind1a-5	564	Hypothetical protein	*Vibrio cholerae*; WP_054104207.1	99
	Ga0061065_11933	mfuind1a-6	912	Protein of unknown function	*Vibrio cholerae*; WP_054104208.1	99
HS-1 (*traJ*-*traL*)	Ga0061065_11928	mfuind1a-7	588	Hypothetical protein	*Shewanella decolorationis*; WP_023266690.1	98
	Ga0061065_11927	mfuind1a-8	240	Hypothetical protein	*Vibrio parahaemolyticus*; WP_025611035.1	96
HS-2 (*traA*-*s054*)	Ga0061065_11920	mfuind1a-9	507	Acetyltransferase domain containing protein	*Vibrio parahaemolyticus*; WP_023584043.1	95
	Ga0061065_11919	mfuind1a-10	267	Uncharacterized conserved protein, DUF1778 family	*Vibrio cholerae*; WP_032481064.1	95
	Ga0061065_11918	mfuind1a-11	2730	Heavy metal transporter CzcA	*Vibrio cholerae*; WP_001901413.1	99
	Ga0061065_11917	mfuind1a-12	1110	Hypothetical protein	*Vibrio parahaemolyticus*; WP_029855131.1	99
	Ga0061065_11916	mfuind1a-13	1533	Phosphatydylserin/phophatydylglycero-phosphate/cardiolipin synthase	*Vibrio cholera*; WP_001910860.1	99
	Ga0061065_11915	mfuind1a-14	1446	Transcriptional regulator containing AAA-type ATPase and DNA binding domains	*Vibrio cholerae*; WP_000369162.1	99
HS-4 (*s073*-*traF*)	Ga0061065_1197	mfuind1a-15	693	Deoxyribonuclease-1	*Vibrio parahaemolyticus*; WP_025441436.1	83
HS-3 (*traN*-*s063*)	Ga0061065_1332	mfuind1a-16	1065	Hypothetical protein	*Shewanella decolorationis*; WP_023266664.1	99

With few exceptions, many of the proteins encoded by the genes in the hotspot regions of the three ICEs did not showed significant sequence identity with the corresponding proteins encoded by genes present in the five hotspots of the 11 reference SXT/R391 ICEs analyzed (Supplementary Table 2). However, a *mosAT*-like toxin-antitoxin (TA) system was present in the HS-2 of both ICE*Mfu*Ind1a and ICE*Mfu*Ind1b; but it was absent in ICE*Mpr*Chn1, instead a *hipAB*-like TA system was present between the *attL* and *xis*, a feature also present in R391 and ICE*Vch*Mex1 (Wozniak et al., 2009; Carraro et al., 2015). Additionally, BLASTX search against the NCBI-RefSeq and the ICEberg (Bi et al., 2012) databases showed that most of the genes in these hotspots encoded proteins similar to those found in other marine *Gammaproteobacteria* (Tables 1–**3**, Supplementary Tables 3–5). The variable genes present in the hotspots of ICE*Mfu*Ind1a and ICE*Mpr*Chn1 showed high sequence identity with known proteins from marine bacteria, whereas those in ICE*Mfu*Ind1b mostly showed relative low sequence identities or were distantly related to known proteins.

Our analysis with the contents of hotspots in ICE*Mfu*Ind1a predicts recombination of genes between the SXT- and R391- related ICEs, while those in ICE*Mfu*Ind1b predict acquisition of genes from unrelated donor cells. More specifically, the HS-2 in ICE*Mfu*Ind1a was composed of genes that encoded proteins with >95% identity with those encoded by ICEs of the SXT group (*mfuind1a-11* to *mfuind1a-14*) and R391-related ICEs (*mfuind1a-9* and *mfuind1a-10*), whereas the HS-2 in ICE*Mfu*In1b was composed of genes that encoded proteins with >92% identity with those encoded by R391 and ICE*Vch*Mex1 (belonging to the R391 group). This suggests the HS-2 of ICE*Mfu*Ind1a is shaped by recombination between SXT- and R391-related ICEs in ICE*Mfu*Ind1a which was also observed by Osorio et al. (2008), whereas the HS-2 of ICE*Mfu*Ind1b was shaped by direct acquisition from a R391-related ancestor. However, the nature and functional attributes of most of these variable hotspot genes are not clear; either they confer element-specific properties or encode functions that have not been described in any known SXT/R391 ICEs. The genes in the hotspots of ICE*Mfu*Ind1a (Table 1) encode proteins which likely protect the host cell from heavy metals toxicity (*mfuind1a-11*), invasion by foreign DNA (*mfuind1a-1* to *mfuind1a-4*) and/or promote integrity of the ICE genome (*mfuind1a-9, mfuind1a-10* and *mfuind1a-15*). Further, the hotspot gene clusters *mfuind1a-1* to *mfuind1a-4* and *mfuind1a-11* to *mfuind1a-14* in ICE*Mfu*Ind1a showed very high similarity to such clusters in the genomes of *Psychromonas arctica* DSM 14288 and *Vibrio cholerae* O1 Inaba G4222 (Table 1). On the other hand, genes present in the hotspots of ICE*Mfu*Ind1b encoded distantly related proteins. The HS-5 of ICE*Mfu*Ind1b (Table 2) contains genes which likely encode proteins related to the functions of aromatic aldehyde oxidation (*mfuind1b-2*), cholesterol degradation (*mfuind1b-3*), histidine degradation by the Hut pathway (*mfuind1b-5* to *mfuind1b-8*), a type-III restriction and modification system (*mfuind1b-11* and *mfuind1b-12*). In addition, genes in HS-3 of ICE*Mfu*Ind1b encoded a HipAB-like toxin-antitoxin system (*mfuind1b-21* and *mfuind1b-22*) involved in either ICE maintenance by killing or severely inhibiting the growth of cells that have lost the element (Wozniak and Waldor, 2009) or persister cells formation (Germain et al., 2013), and a predicted novel chemotaxis signal transduction system (*mfuind1b-23* to *mfuind1b-25*). Although ICEs of the SXT/R391 family are well known for fostering dissemination of multidrug resistance genes in both environmental and clinical isolates (Wozniak et al., 2009; Carraro and Burrus, 2014; Spagnoletti et al., 2014; Johnson and Grossman, 2015), interestingly no such genes are found in either ICE*Mfu*Ind1a or ICE*Mfu*Ind1b of the *M. fungiae* JCM 18476^T^. However, analysis of the hotspot regions of ICE*Mpr*Chn1 (Table 3) revealed the HS-1 contained a putative transposon cassette with genes conferring possible multidrug resistance phenotype to the host *M. profundimaris* (*mprchn1*-6 to *mprchn1*-18). Additionally, the HS-2 also bear a gene predicted to confer heavy metal resistance (*mprchn*1-24), whereas the HS-5 bear a putative helicase and a type-III restriction and modification system (*mprchn1-3* to *mprchn1-5*). Overall, these hotspot variable genes likely encode functional traits that are advantageous to the host in changing environments and/or for stable maintenance of the ICEs.

**Table 2:** **Descriptions of genes or ORFs present in the hotspot regions of ICE*Mfu*Ind1b**.

**Hotspot region**	**ICE*Mfu*Ind1b gene/ORF id**	**Gene name**	**Length (bp)**	**Hotspot gene product name**	**GenBank accession no. of protein homolog**	**% Similarity with homolog**
HS-5 (*s024*-*traI*)	Ga0061065_12215	mfuind1b-1	1038	AraC-type DNA-binding protein	*Acinetobacter tandoii*; WP_016166436.1	49
	Ga0061065_12214	mfuind1b-2	1485	Coniferyl-aldehyde dehydrogenase	*Vibrio litoralis*; WP_027695211.1	75
	Ga0061065_12213	mfuind1b-3	1779	Cholesterol oxidase	*Vibrio litoralis*; WP_027695212.1	69
	Ga0061065_12212	mfuind1b-4	900	DNA-binding transcriptional regulator, LysR	*Colwellia piezophila*; WP_019026485.1	75
	Ga0061065_12211	mfuind1b-5	1512	Histidine ammonia lyase	*Pseudoalteromonas tunicate*; WP_009838609.1	84
	Ga0061065_12210	mfuind1b-6	2013	Urocanate hydratase	*Pseudoalteromonas luteoviolacea*; WP_023399981.1	92
	Ga0061065_1229	mfuind1b-7	1272	Imidazolonepropionase	*Oceanospirillum beijerinckii*; WP_028302424.1	86
	Ga0061065_1228	mfuind1b-8	1050	Formiminoglutamase	*Oceanospirillum beijerinckii*; WP_028302423.1	68
	Ga0061065_1227	mfuind1b-9	240	Hypothetical protein	No homology	–
	Ga0061065_1226	mfuind1b-10	1254	Hypothetical protein	*Oceanospirillum beijerinckii*; WP_028301959.1	47
	Ga0061065_1225	mfuind1b-11	885	DNA methylase	*Vibrio parahaemolyticus*; WP_023584030.1	77
	Ga0061065_1224	mfuind1b-12	3078	Type-III restriction enzyme	*Vibrio parahaemolyticus*; WP_023584031.1	99
	Ga0061065_1223	mfuind1b-13	282	NMD3 family protein	*Vibrio parahaemolyticus*; WP_023584032.1	98
	Ga0061065_1222	mfuind1b-14	474	Hypothetical protein	*Vibrio cholerae*; WP_001218616.1	100
HS-1 (*traJ*-*traL*)	Sequence not known		–	–	–	–
HS-2 (*traA*-*s054*)	Ga0061065_1236	mfuind1b-15	507	Acetyltransferase (GNAT) domain containing protein	*Vibrio parahaemolyticus*; WP_025586955.1	98
	Ga0061065_1237	mfuind1b-16	267	Uncharacterized conserved protein DUF1778 family	*Vibrio*; WP_000212004.1	100
HS-4 (*s073*-*traF*)	Ga0061065_12315	mfuind1b-17	684	Hypothetical protein	*Escherichia coli*; WP_001375061.1	52
	Ga0061065_12316	mfuind1b-18	897	Protein of unknown function DUF4433	*Pseudomonas syringae*; WP_024639153.1	41
	Ga0061065_12317	mfuind1b-19	1131	TPR repeat containing protein	*Denitrovibrio acetiphilus*; WP_013010018.1	26
	Ga0061065_12318	mfuind1b-20	531	Hypothetical protein	*Acinetobacter baumannii*; WP_004742094.1	35
	Ga0061065_12319	mfuind1b-21	1317	Serine/threonine protein kinase HipA	*Vibrio vulnificus*; WP_011080066.1	95
	Ga0061065_12320	mfuind1b-22	324	Cro/C1-type transcriptional regulator	*Vibrio vulnificus*; WP_017790219.1	92
HS-3 (*traN*-*s063*)	Ga0061065_1306	mfuind1b-23	1782	PAS domain S-box containing protein	*Nitrosomonas* sp.; WP_013965400.1	35
	Ga0061065_1305	mfuind1b-24	2400	PAS domain S-box containing protein	*Rheinheimera nanhaiensis*; WP_008221817.1	44
	Ga0061065_1304	mfuind1b-25	1557	Methyl accepting chamotaxis sensory transducer with Pas/Pac sensor	*Vibrio furnissii*; WP_004729586.1	59

**Table 3:** **Descriptions of genes or ORFs present in the hotspot regions of ICE*Mpr*Chn1**.

**Hotspot region**	**ICE*MprChn1* gene/ORF id**	**Gene name**	**Length (bp)**	**Hotspot gene product name**	**GenBank accession no. of protein homolog**	**% Similarity with homolog**
HS-5 (*s026*-*traI*)	D104_09935	mprchn1-1	3333	DNA or RNA helicase	*Salmonella enterica*; WP_060636796.1	100
	D104_09940	mprchn1-2	687	Hypothetical protein	*Salinivibrio socompensis*; WP_025674120.1	99
	D104_09945	mprchn1-3	1947	DNA methyltransferase	*Pseudomonas aeruginosa*; WP_034019474.1	99
	D104_09950	mprchn1-4	3078	Type III restriction enzyme R subunit	*Shewanella decolorationis*; WP_023266696.1	99
	D104_09955	mprchn1-5	855	Restriction endonuclease	*Vibrio fluvialis*; WP_052075075.1	98
HS-1 (*traJ*-*traL*)	D104_09980	mprchn1-6	1557	Transposase	*Gammaproteobacteria*; WP_000850406.1	100
	D104_09985	mprchn1-7	96	Hypothetical protein	–	N.h
	D104_09995	mprchn1-8	219	Hypothetical protein	*Gammaproteobacteria*; WP_013785946.1	99
	D104_10000	mprchn1-9	243	Hypothetical protein	*Nitrincola* sp. *A-D6*; WP_036520787.1	98
	D104_10005	mprchn1-10	708	RES domain-containing protein	*Nitrincola nitratireducens*; WP_036513282.1	67
	D104_10010	mprchn1-11	372	Hypothetical protein	*Neptunomonas Antarctica*; WP_054341548.1	79
	D104_10015	mprchn1-12	693	TetR family transcriptional regulator	*Alteromonas* sp. *SN2*; WP_013785950.1	99
	D104_10020	mprchn1-13	1047	RND transporter	*Marinobacter* sp. *CP1*; WP_053115257.1	54
	D104_10025	mprchn1-14	3042	Multidrug transporter	*Alteromonas* sp. *SN2*; WP_013785952.1	99
	D104_10030	mprchn1-15	615	SAM-dependent methyltransferase	*Nitrincola* sp. A-D6; WP_036520790.1	72
	D104_10035	mprchn1-16	234	Hypothetical protein	*Nitrincola* sp. A-D6; WP_052063520.1	88
	D104_10040	mprchn1-17	1305	Transposase	*Shewanella putrefaciens*; WP_014611283.1	74
	D104_10445	mprchn1-18	1173	Transposase	*Alteromonas* sp. *SN2*; WP_041452603.1	98
	D104_10450	mprchn1-19	696	ATPase AAA	*Vibrio cholerae*; WP_000796550.1	99
	D104_10455	mprchn1-20	573	Plasmid-related protein	*Alteromonas macleodii*; WP_061094531.1	96
HS-2 (*traA*-*s054*)	D104_10490	mprchn1-21	834	Hypothetical protein	*Vibrio nigripulchritudo*; WP_022609808.1	99
	D104_10495	mprchn1-22	702	Hypothetical protein	*Vibrio cholerae*; WP_053044234.1	99
	D104_10500	mprchn1-23	408	MerR family transcriptional regulator	*Serratia marcescens*; YP_003602521.1	100
	D104_10505	mprchn1-24	885	Sodium: proton antiporter, CzcD	*Pseudoalteromonas shioyasakiensis*; WP_063529117.1	99
HS-4 (*s073*-*traF*)	D104_04195	mprchn1-25	434	Hypothetical protein	*Vibrio alginolyticus*; WP_005396849.1	97
	D104_04190	mprchn1-26	663	Hypothetical protein	*Vibrio alginolyticus*; WP_005396850.1	100

N.h, No homology.

## Conclusions

Our analysis showed all the three ICEs shared a similar genetic organization with SXT/R391-like ICEs. In the backbone most of the syntenic core genes are conserved. Further, the two ICEs of *M. fungiae* were inserted at two different sites in the genome. Our analysis suggested the conjugative transfer of a second copy of SXT ICE in the same cell is not always impeded by the typical TraG/Eex mediated entry exclusion mechanism. Hotspots regions of all the three ICEs showed presence of large numbers of unique variable genes which were not found in the previously described ICEs. Further, absence of multidrug resistance genes in the hotspots suggested ICEs of *M. fungiae* have probably evolved through homologous recombination; in contrast the ICE*Mpr*Chn1 of *M. profundimaris* strain D104 harbored a typical transposon cassette with multidrug transporter genes in the HS-1. The existence of such ICEs in marine bacteria warrants their rapid identification and functional analysis to understand the dissemination of multidrug resistance genes and their impact in natural populations.

## Author contributions

SD conceived the idea of the work. JB designed the experiments and performed the experiments. JB and SD analyzed the data and wrote the manuscript.

### Conflict of interest statement

The authors declare that the research was conducted in the absence of any commercial or financial relationships that could be construed as a potential conflict of interest.
