# Supplementary material for: Characterization of Three Novel SXT/R391 Integrating Conjugative Elements ICEMfuInd1a and ICEMfuInd1b, and ICEMprChn1 Identified in the Genomes of Marinomonas fungiae JCM 18476T and Marinomonas profundimaris Strain D104
Source: Front Microbiol. 2016 Nov 25;7:1896. doi: 10.3389/fmicb.2016.01896 (PMC5122569; doi:10.3389/fmicb.2016.01896)
Supplement: Supplementary file 1 [file DataSheet1.pdf]

SUPPLEMENTARY MATERIALS

Characterization of three novel SXT/R391 integrating conjugative elements ICE*Mfu*Ind1a and ICE*Mfu*Ind1b, and ICE*Mpr*Chn1 identified in the genomes of *Marinomonas fungiae* JCM 18476^T and *Marinomonas profundimaris* strain D104.

Journal: Frontiers in Microbiology

Authors: Jhasketan Badhai and Subrata K Das*

Addresses: Department of Biotechnology, Institute of Life Sciences, Nalco Square, Bhubaneswar, India.

***Correspondence:**
Institute of Life Sciences
Department of Biotechnology
Nalco Square, Bhubaneswar 751023 India
E-mail: subratkdas@hotmail.com; subrata@ils.res.in
Phone: (+91) 674 230 3342
Fax: (+91) 674 230 0728

```
STXMO10        GCT CTG TTT GGC GGC GGA TGA CCT AGT CAA AAA AAT CGA GAC GCC AAA CGT CGT TTG CAT TCT GGC CTG AAC TCG CCA
ICEVchInd4     ... ... ... ... ... ... ... ... ... ... ... ... ... ... ... ... ... ... ... ... ... ... ... ... ... ...
ICEPdaSpa1     ... ... ... ... ... ... ... ... ... ... ... ... ... ... ... ... ... ... ... ... ... ... ... ... ... ...
ICEPmiChn1     ... ... ... ... ... T.G ... ... ..C ... ... ... T.. ... ... ... ... ... ... ... ... ... ... ... .T. ...
ICEMfuInd1b    ... ... ... ... ... ... .A. ... ..C ... ... ... ... ... ... ... ... ... ... ... ... ... ... ... .T. ...
ICESpuPO1      ..G ... ... ... ... ... ... .TG ..C ... ... ... ... ... ... ... ... ... ... ... ... ... ... ... ... ...
ICEVflInd1     ... ... ... ... ... T.G ... ... ..C ... ... ... T.. ... ... ... ... ... ... ... ... ... ... ... .T. ...
ICEMprChn1     ... ... ... ... ... ... .A. .TG ..C ... ... ... ... ... ... ... T.. ... ... ... ... ... ... ... ... ...
R391           ... ... ... ... ..T ... ... .TG ..C ... ... ... ... ... ... ... ... ... ... ... ... ... ... ... ... ...
ICEVchMex1     ... ... ... ... ... ... ... ... ... ... ... ... ... ... ... ... ... ... ... ... ... ... ... ... ... ...
ICEPalBan1     ... ... ... ... ... T.G ... ... ..C ... ... ... T.. ... ... ... ... ... ... ... ... ... ... ... .T. ...
ICEVchBan5     ... ... ... ... ... T.G ... ... ..C ... ... ... T.. ... ... ... ... ... ... ... ... ... ... ... .T. ...
ICEVchInd5     ... ... ... ... ... T.G ... ... ..C ... ... ... T.. ... ... ... ... ... ... ... ... ... ... ... .T. ...
ICEMfuInd1a    ... ... ... ... ... ... ... ... ... ... ... ... ... ... ... ... ... ... ... ... ... ... T.. ... .T. ...

STXMO10        AAC GGT TTG TAT CTT CAT GAC GAT ACG TCG TTT TAG GCG TTT TTA AGT GAA ATC GGG CTG TAT CCC TTG TCA GGT ATG
ICEVchInd4     ... ... ... ... ... ... ... ... ... ... ... ... ... ... ... ... ... ... ... ... ... ... ... ... ... ...
ICEPdaSpa1     ... ... ... ... ... ... ... ... ... ... ... ... ... ... ... ... ... ... ... ... ... ... ... ... ... ...
ICEPmiChn1     ... ... ... ... ... ... .G. ... ... ..T ... ... .T. ... ... ... ... ... A.C ... ... ... ... ..G ... ...
ICEMfuInd1b    ... ... ... ... T.. ... .G. A.. ... ..T ... ... ... ... ... ... ... ... ..C ... C.. ... ... ... ... ...
ICESpuPO1      ... ... ... ... ... ... ... ... ... ..T ... ... ... ... ... ... ... ... ... ... ... ... ... ... ... ...
ICEVflInd1     ... ... ... ... ... ... .G. ... ... ..T ... ... .T. ... ... ... ... ... A.C ... ... ... ... ..G ... ...
ICEMprChn1     ... ... ... ... ... ... ... ... ... ..T ... ... ... ... ... ... ... ... ... ... ... ... ... ... ... ...
R391           ... ... ... ... ... ... .G. ... ... ..T ... C.. ..A A.. T.. ... ... ... ... ... ... ... ... ... ... ...
ICEVchMex1     ... ... ... ... ... ... ... ... ... ... ... ... ... ... ... ... T.. ... ... ... ... ... ... ... ... ...
ICEPalBan1     ... ... ... ... ... ... .G. ... ... ..T ... ... .T. ... ... ... ... ... A.C ... ... ... ... ..G ... ...
ICEVchBan5     ... ... ... ... ... ... .G. ... ... ..T ... ... .T. ... ... ... ... ... A.C ... ... ... ... ..G ... ...
ICEVchInd5     ... ... ... ... ... ... .G. ... ... ..T ... ... .T. ... ... ... ... ... A.C ... ... ... ... ..G ... ...
ICEMfuInd1a    ... ... ... ... T.. ... .G. ... ... ..T ... ... ... ... ..C ... ... ... ..C ... ... ... ... ... ... ...

STXMO10        GGA TTG CGC GAG TTG ATT TAT ATC GAG ACG CCA AAC AGT GAT TGT TAC GGC AGT TTT ACG TTT GGC GTT TCG ATC CAA
ICEVchInd4     ... ... ... ... ... ... ... ... ... ... ... ... ... ... ... ... ... ... ... ... ... ... ... ... ... ...
ICEPdaSpa1     ... ... ... ... ... ... ... ... ... ... ... ... ... ... ... ... ... ... ... ... ... ... ... ... ... ...
ICEPmiChn1     ... ... A.. ... .C. ... ... ... ... ... ... ... ... ... ... G.. ... ... ... ... ... ... ... ... ... ...
ICEMfuInd1b    ... ... ... ... ... ... ... ... ... ... ... ... ... ... ... ... ... ... ... ... ... ... ... ... ... ...
ICESpuPO1      ... ... ... ... ... ... ... ... ... ... ... ... ... ... ... G.. ..T ... ... ... ... ... ... ... ... ...
ICEVflInd1     ... ... A.. ... .C. ... ... ... ... ... ... ... ... ... ... G.. ... ... ... ... ... ... ... ... ... ...
ICEMprChn1     ..G ... ... ... ... ... ... ... ... ... ... ... ... ... ... ... C.. ... ... ... ... ... ... ... ... ...
R391           ... ... ... ... ... ... ... ... ... ... ... ... ... ... ... ... ... ... ... ... ... ... ... ... ... ...
ICEVchMex1     ... ... ... ... ... ... ... ... ... ... ... ... ... ... ... ... ... ... ... ... ... ... ... ... ... ...
ICEPalBan1     ... ... A.. ... .C. ... ... ... ... ... ... ... ... ... ... G.. ... ... ... ... ... ... ... ... ... ...
ICEVchBan5     ... ... A.. ... .C. ... ... ... ... ... ... ... ... ... ... G.. ... ... ... ... ... ... ... ... ... ...
ICEVchInd5     ... ... A.. ... .C. ... ... ... ... ... ... ... ... ... ... G.. ... ... ... ... ... ... ... ... ... ...
ICEMfuInd1a    ... ... ... ... ... ... ... ... ... ... ... ... ... ... ... ... ... ... ... ... ... ... ... ... ... ...

STXMO10        AAG CCA AAC GGA TAG TGG TTT TGG CTT TTG GGG TTA ATT GGA TGG GGA AAT TGG TTT GGT AGA AA
ICEVchInd4     ... ... ... ... ... ... ... ... ... ... ... ... ... ... ... ... ... ... ... ... ... ..
ICEPdaSpa1     ... ... ... ... ... ... ... ... ... ... ... ... ... ... ... ... ... ... ... ... ... ..
ICEPmiChn1     ... ... ... ... ... ... ... ... ... .A. ... ... ... ... ... ... ... ... ... ... ... ..
ICEMfuInd1b    ... ... ... ... ... ... ... ... ... ... ..T ... ... ... ... ... ... ... ... ... ... ..
ICESpuPO1      ... ... ... ... ... ... ... ... ... ... ... ... ... ..G ..A ... ... ... ... ... ... ..
ICEVflInd1     ... ... ... ... ... ... ... ... ... .A. ... ... ... ... ... ... ... ... ... ... ... ..
ICEMprChn1     ... ... ... ... ... ... ... ... ... ... ... ... ... ... ... ... ... ... ... ... ... ..
R391           ... ... ... ... ... .A. ... ... T.. ... ... ... ... ... ... ... ... ... ... ... ... ..
ICEVchMex1     ... ... ... ... ... ... ... ... ... ... ... ... ... ... ... ... ... ... ... ... ... ..
ICEPalBan1     ... ... ... ... ... ... ... ... ... .A. ... ... ... ... ... ... ... ... ... ... ... ..
ICEVchBan5     ... ... ... ... ... ... ... ... ... .A. ... ... ... ... ... ... ... ... ... ... ... ..
ICEVchInd5     ... ... ... ... ... ... ... ... ... .A. ... ... ... ... ... ... ... ... ... ... ... ..
ICEMfuInd1a    ... ... ... ... ... ... ... ... ... ... ... ... ... ... ... ... ... ... ... ... ... ..
```

Fig. S1

Supplementary Figure S1. ClustalW alignment on MEGA6 of the 299-bp long putative *oriT* DNA sequence located within the intergenic region *srpM-mobI* in ICE*Mfu*Ind1a, ICE*Mfu*Ind1b and ICE*Mpr*Chn1 with the corresponding sequences from 11 reference SXT/R391 ICEs. Reference sequences were retrieved from the GenBank: SXT^{MO10} (*V. cholerae* O139; accession: AY055428), ICE*Vch*Ind4 (*V. cholerae* O139; accession: GQ463141), ICE*Vch*Ind5 (*V. cholerae* O1; accession: GQ463142), ICE*Vch*Ban5 (*V. cholerae* O1; accession: GQ463140), ICE*Vch*Mex1 (*V. cholerae* non O139; accession: GQ463143), R391 (*Providencia rettgeri*; accession: AY090559), ICE*Pal*Ban1 (*Providencia alcalifaciens*; accession: GQ463139), ICE*Vfl*Ind1 (*V. fluvialis*; accession: GQ463144), ICE*Pda*Spa1 (*Photobacterium damselae*; accession: AJ870986), ICE*Spu*PO1 (*Shewanella putrefaciens*; accession: CP000503), and ICE*Pmi*Chn1 (*Proteus mirabilis*; accession: KT962845).

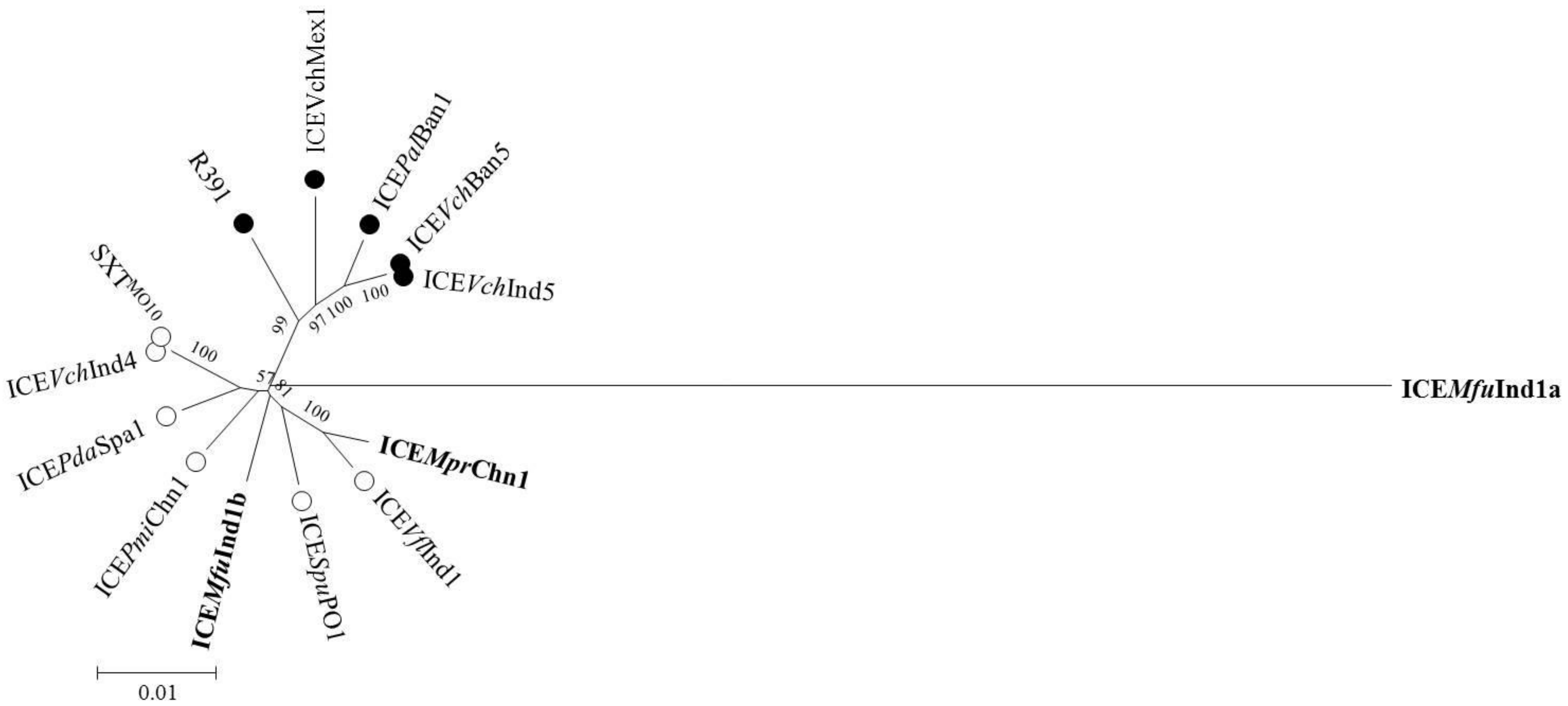

Supplementary Figure S2. Phylogenetic analysis of core ICE genes encoded proteins. The unrooted tree was constructed by applying the Maximum Likelihood method based on the Poisson correction model using the MEGA6. Bootstrap analysis with 1000 replications was performed to test the reliability of the tree. (●), ICEs of the R391 exclusion group; (○), ICEs of the SXT exclusion group.

Supplementary Table S1. Predicted core genes or ORFs present in the SXT-like ICEs: **(A)**, ICE*Mfu*Ind1a and **(B)**, ICE*Mfu*Ind1b derived from the genome of *M. fungiae* JCM 18476^{T}; and (**C**), ICE*Mpr*Chn1 of *M. profundimaris* strain D104.

(A) Core genes in ICE*Mfu*Ind1a

ICE*Mfu*Ind1a gene/ORF id	Putative gene product name	SXT/R391 equivalent gene	GenBank accession no. of *M. fungiae* genomic scaffolds encoding the ICE
Ga0061065_11949	Transcriptional regulator, AlpA family	*xis*	LIQF01000019.1
Ga0061065_11948[*]	Integrase, N-terminal region	*int*	LIQF01000019.1
Ga0061065_11947[*]	Integrase	*int*	LIQF01000019.1
Ga0061065_11946	Hypothetical protein	*srpR*	LIQF01000019.1
Ga0061065_11945	Plasmid segregation protein	*srpM*	LIQF01000019.1
Ga0061065_11944	Hypothetical protein	*mobI*	LIQF01000019.1
Ga0061065_11943	DNA PolV/UmuCDNA repair protein	*rumB*	LIQF01000019.1
Ga0061065_11942	DNA PolV/UmuDDNA repair protein	*rumA*	LIQF01000019.1
Ga0061065_11941	DNA polymerase-3 subunit epsilon	*s024*	LIQF01000019.1
Ga0061065_11940	Hypothetical protein	*s025*	LIQF01000019.1
Ga0061065_11939	WYL domain containing protein	*s026*	LIQF01000019.1
Ga0061065_11932	Conjugal transfer pilus assembly protein	*traI*	LIQF01000019.1
Ga0061065_11931	Conjugal transfer pilus assembly protein	*traD*	LIQF01000019.1
Ga0061065_11930	Conjugal transfer pilus assembly protein	*s091*	LIQF01000019.1
Ga0061065_11929	Conjugal transfer pilus assembly protein	*s043*/*traJ*	LIQF01000019.1
Ga0061065_11926	Conjugal transfer pilus assembly protein	*traL*	LIQF01000019.1
Ga0061065_11925	Conjugal transfer pilus assembly protein	*traE*	LIQF01000019.1
Ga0061065_11924	Conjugal transfer pilus assembly protein	*traK*	LIQF01000019.1
Ga0061065_11923	Conjugal transfer pilus assembly protein	*traB*	LIQF01000019.1
Ga0061065_11922	Conjugal transfer pilus assembly protein	*traV*	LIQF01000019.1
Ga0061065_11921	Conjugal transfer pilus assembly protein	*traA*	LIQF01000019.1
Ga0061065_11914	Thiol:disulfide interchange protein	*s054*/*dsbC*	LIQF01000019.1
Ga0061065_11913	Type-IV secretion system protein	*traC*	LIQF01000019.1
Ga0061065_11912	Conserved hypothetical protein	*orf92*	LIQF01000019.1
Ga0061065_11911	Conjugal transfer pilin signal peptidase	*trhF*	LIQF01000019.1
Ga0061065_11910	Conjugal transfer pilus assembly protein	*traW*	LIQF01000019.1
Ga0061065_1199	Conjugal transfer pilus assembly protein	*traU*	LIQF01000019.1
Ga0061065_1198	Conjugal transfer pilus assembly protein	*traN*	LIQF01000019.1
Ga0061065_1196	Hypothetical protein	*s063*	LIQF01000019.1

ICE*Mfu*Ind1a gene/ORF id	Putative gene product name	SXT/R391 equivalent gene	GenBank accession no. of *M. fungiae* genomic scaffolds encoding the ICE
Ga0061065_1195	Hypothetical protein	*s089*	LIQF01000019.1
Ga0061065_1194	Single strand binding protein	*ssb*	LIQF01000019.1
Ga0061065_1193	Phage recombination protein	*bet*	LIQF01000019.1
Ga0061065_1192	Putative phage-type endonuclease	*exo*	LIQF01000019.1
Ga0061065_1191	AAA-ATPase domain, CobS	*s067*	LIQF01000019.1
Ga0061065_1331	Hypothetical protein	*s073*	LIQF01000033.1
Ga0061065_1333	Conjugal transfer pilus assembly protein	*traF*	LIQF01000033.1
Ga0061065_1334	Conjugal transfer pilus assembly protein	*traH*	LIQF01000033.1
Ga0061065_1335*	TraG-like protein, N-terminal region	*traG*	LIQF01000033.1
Ga0061065_11487*	Conjugal transfer mating pair stabilization protein	*traG*	LIQF01000033.1
Ga0061065_11486	Entry exclusion protein	*eex*	LIQF01000014.1
Ga0061065_11485	Flagellar transcriptional activator (FlhC)	*setC*	LIQF01000014.1
Ga0061065_11484	Flagellar transcriptional activator (FlhD)	*setD*	LIQF01000014.1
Ga0061065_11483	Transglycosylase SLT domain containing protein	*s082*	LIQF01000014.1
Ga0061065_11482	Hypothetical protein	*s083*	LIQF01000014.1
Ga0061065_11481	Hypothetical protein	*s084*	LIQF01000014.1
Ga0061065_11480	Putative antitoxin of toxin-antitoxin system	*croS*/*setQ*	LIQF01000014.1
Ga0061065_11479	Phage repressor protein	*setR*	LIQF01000014.1

(B) Core genes in ICE*Mfu*Ind1b

ICE*Mfu*Ind1b gene/ORF id	Putative gene product name	SXT/R391 equivalent gene	GenBank accession no. of *M. fungiae* genomic scaffolds encoding the ICEs
Ga0061065_12222	Integrase	*int*	LIQF01000022.1
Ga0061065_12221	Hypothetical protein	*srpR*	LIQF01000022.1
Ga0061065_12220	Plasmid segregation protein	*srpM*	LIQF01000022.1
Ga0061065_12219	Hypothetical protein	*mobI*	LIQF01000022.1
Ga0061065_12218	DNA PolV/UmuCDNA repair protein	*rumB*	LIQF01000022.1
Ga0061065_12217	DNA PolV/UmuDDNA repair protein	*rumA*	LIQF01000022.1
Ga0061065_12216	Hypothetical protein	*s024*	LIQF01000022.1
Ga0061065_1221*	Conjugal transfer pilus assembly protein	*traI*	LIQF01000022.1
Ga0061065_1231*	Conjugal transfer pilus assembly protein	*traE*	LIQF01000023.1
Ga0061065_1232	Conjugal transfer pilus assembly protein	*traK*	LIQF01000023.1
Ga0061065_1233	Conjugal transfer pilus assembly protein	*traB*	LIQF01000023.1
Ga0061065_1234	Conjugal transfer pilus assembly protein	*traV*	LIQF01000023.1

ICE*Mfu*Ind1b gene/ORF id	Putative gene product name	SXT/R391 equivalent gene	GenBank accession no. of *M. fungiae* genomic scaffolds encoding the ICEs
Ga0061065_1235	Conjugal transfer pilus assembly protein	*traA*	LIQF01000023.1
Ga0061065_1238	Thiol:disulfide interchange protein	*s054/dsbC*	LIQF01000023.1
Ga0061065_1239	Type-IV secretion system protein	*traC*	LIQF01000023.1
Ga0061065_12310	Conserved hypothetical protein	*orf92*	LIQF01000023.1
Ga0061065_12311	Conjugal transfer pilin signal peptidase	*trhF*	LIQF01000023.1
Ga0061065_12312	Conjugal transfer pilus assembly protein	*traW*	LIQF01000023.1
Ga0061065_12313	Conjugal transfer pilus assembly protein	*traU*	LIQF01000023.1
Ga0061065_12314	Conjugal transfer pilus assembly protein	*traN*	LIQF01000023.1
Ga0061065_12321	Hypothetical protein	*s063*	LIQF01000023.1
Ga0061065_12322	Hypothetical protein	*s089*	LIQF01000023.1
Ga0061065_12323	Single strand binding protein	*ssb*	LIQF01000023.1
Ga0061065_12324	Phage recombination protein	*bet*	LIQF01000023.1
Ga0061065_12325	Hypothetical protein	*orfZ*	LIQF01000023.1
Ga0061065_12326	Putative phage-type endonuclease	*exo*	LIQF01000023.1
Ga0061065_12327	Cobaltochelatase, CobS	*s067*	LIQF01000023.1
Ga0061065_12328	Hypothetical protein	*s088*	LIQF01000023.1
Ga0061065_13011	von Willebrand factor type-A domain containing protein	*s070*	LIQF01000030.1
Ga0061065_13010	RadC-like JAB domain	*s071*	LIQF01000030.1
Ga0061065_1309	Hypothetical protein	*s092*	LIQF01000030.1
Ga0061065_1308	Uncharacterized domain associated with phage or plasmid primase	*s072*	LIQF01000030.1
Ga0061065_1307	Hypothetical protein	*s073*	LIQF01000030.1
Ga0061065_1303	Conjugal transfer pilus assembly protein	*traF*	LIQF01000030.1
Ga0061065_1302	Conjugal transfer pilus assembly protein	*traH*	LIQF01000030.1
Ga0061065_1301[*]	TraG-like protein, N-terminal region	*traG*	LIQF01000030.1
Ga0061065_109135[*]	Conjugal transfer mating pair stabilization protein	*traG*	LIQF01000009.1
Ga0061065_109134	Entry exclusion protein	*eex*	LIQF01000009.1
Ga0061065_109133	Flagellar transcriptional activator (FlhC)	*setC*	LIQF01000009.1
Ga0061065_109132	Flagellar transcriptional activator (FlhD)	*setD*	LIQF01000009.1
Ga0061065_109131	Transglycosylase SLT domain containing protein	*s082*	LIQF01000009.1
Ga0061065_109130	Hypothetical protein	*s083*	LIQF01000009.1
Ga0061065_109129	Hypothetical protein	*s084*	LIQF01000009.1
Ga0061065_109128	Putative antitoxin of toxin-antitoxin system	croS/setQ	LIQF01000009.1
Ga0061065_109127	Phage repressor protein	*setR*	LIQF01000009.1

(C) Core genes in ICE*Mpr*Chn1

ICE*Mpr*Chn1 gene/ORF id	Putative gene product name	SXT/R391 equivalent gene	GenBank accession no. of *M. profundimaris* D104 genomic scaffolds encoding the ICEs
D104_12225	Cro/C1-type helix-turn-helix domain	*hipB*	AYOZ01000034.1
D104_12220	Serine/threonine-protein kinase HipA	*hipA*	AYOZ01000034.1
D104_12210	Transcriptional regulator, AlpA family	*xis*	AYOZ01000034.1
D104_12205	Integrase	*int*	AYOZ01000034.1
D104_12200	Hypothetical protein	*srpR*	AYOZ01000034.1
D104_12195	Plasmid segregation protein	*srpM*	AYOZ01000034.1
D104_12190	Hypothetical protein	*mobI*	AYOZ01000034.1
D104_12185	DNA PolV/UmuCDNA repair protein	*rumB*	AYOZ01000034.1
D104_12180	DNA PolV/UmuCDNA repair protein, N-terminal	*rumB*	AYOZ01000034.1
D104_12175	DNA PolV/UmuDDNA repair protein	*rumA*	AYOZ01000034.1
D104_09920	DNA polymerase-3 subunit epsilon	*s024*	AYOZ01000017.1
D104_09925	Hypothetical protein	*s025*	AYOZ01000017.1
D104_09930	WYL domain containing protein	*s026*	AYOZ01000017.1
D104_09960	Conjugal transfer pilus assembly protein	*traI*	AYOZ01000017.1
D104_09965	Conjugal transfer pilus assembly protein	*traD*	AYOZ01000017.1
D104_09970	Conjugal transfer pilus assembly protein	*s091*	AYOZ01000017.1
D104_09975	Conjugal transfer pilus assembly protein	*s043*/*traJ*	AYOZ01000017.1
D104_10460	Conjugal transfer pilus assembly protein	*traL*	AYOZ01000022.1
D104_10465	Conjugal transfer pilus assembly protein	*traE*	AYOZ01000022.1
D104_10470	Conjugal transfer pilus assembly protein	*traK*	AYOZ01000022.1
D104_10475	Conjugal transfer pilus assembly protein	*traB*	AYOZ01000022.1
D104_10480	Conjugal transfer pilus assembly protein	*traV*	AYOZ01000022.1
D104_10485	Conjugal transfer pilus assembly protein	*traA*	AYOZ01000022.1
D104_04230	Thiol:disulfide interchange protein	*s054/dsbC*	AYOZ01000004.1
D104_04225	Type-IV secretion system protein	*traC*	AYOZ01000004.1
D104_04220	Conserved hypothetical protein	*orf92*	AYOZ01000004.1
D104_04215	Conjugal transfer pilin signal peptidase	*trhF*	AYOZ01000004.1
D104_04210	Conjugal transfer pilus assembly protein	*traW*	AYOZ01000004.1
D104_04205	Conjugal transfer pilus assembly protein	*traU*	AYOZ01000004.1
D104_04200	Conjugal transfer pilus assembly protein	*traN*	AYOZ01000004.1
D104_04185	Hypothetical protein	*s063*	AYOZ01000004.1
D104_04180	Hypothetical protein	*s089*	AYOZ01000004.1
D104_04175	Single strand binding protein	*ssb*	AYOZ01000004.1

ICE*Mpr*Chn1 gene/ORF id	Putative gene product name	SXT/R391 equivalent gene	GenBank accession no. of *M. profundimaris* D104 genomic scaffolds encoding the ICEs
D104_04170	Phage recombination protein	*bet*	AYOZ01000004.1
D104_04165	Hypothetical protein	*orfZ*	AYOZ01000004.1
D104_04160	Putative phage-type endonuclease	*exo*	AYOZ01000004.1
D104_04155	Cobaltochelatase, CobS	*s067*	AYOZ01000004.1
D104_04150	Hypothetical protein	*s088*	AYOZ01000004.1
D104_04145	Protein of unknown function (DUF3150)	*s068*	AYOZ01000004.1
D104_04140	Hypothetical protein	*s069*	AYOZ01000004.1
D104_04135	von Willebrand factor type-A domain containing protein	*s070*	AYOZ01000004.1
D104_04130	RadC-like JAB domain	*s071*	AYOZ01000004.1
D104_04125	Hypothetical protein	*s092*	AYOZ01000004.1
D104_04120	Plasmid primase	*s072*	AYOZ01000004.1
D104_04110	Hypothetical protein	*s073*	AYOZ01000004.1
D104_04105	Conjugal transfer pilus assembly protein	*traF*	AYOZ01000004.1
D104_04100	Conjugal transfer pilus assembly protein	*traH*	AYOZ01000004.1
D104_04095	Conjugal transfer mating pair stabilization protein	*traG*	AYOZ01000004.1
D104_04090	Entry exclusion protein	*eex*	AYOZ01000004.1
D104_04085	Flagellar transcriptional activator (FlhC)	*setC*	AYOZ01000004.1
D104_04080	Flagellar transcriptional activator (FlhD)	*setD*	AYOZ01000004.1
D104_04075	Transglycosylase SLT domain containing protein	*s082*	AYOZ01000004.1
D104_04070	Hypothetical protein	*s083*	AYOZ01000004.1
D104_04065	Hypothetical protein	*s084*	AYOZ01000004.1
D104_04060	Putative antitoxin of toxin-antitoxin system	croS/setQ	AYOZ01000004.1
D104_04055	Phage repressor protein	*setR*	AYOZ01000004.1

SXT/R391 ICE equivalent gene are based on TBLASTN homology search of genes in the two ICEs of *M. fungiae* with the corresponding proteins encoded by the SXT/R391 ICEs.

*, partial sequence.

Supplementary Table S2. Percentage homology of the predicted proteins encoded by the two SXT-like ICEs: **(A)** ICE*Mfu*Ind1a and **(B)** ICE*Mfu*Ind1b derived from *M. fungiae* JCM 18476^T with those of the 11 references SXT/R391 ICEs: SXT^{MO10} (*V. cholerae* O139; accession: AY055428), ICE*Vch*Ind4 (*V. cholerae* O139; accession: GQ463141), ICE*Vch*Ind5 (*V. cholerae* O1; accession: GQ463142), ICE*Vch*Ban5 (*V. cholerae* O1; accession: GQ463140), ICE*Vch*Mex1 (*V. cholerae* non O139; accession: GQ463143), R391 (*Providencia rettgeri*; accession: AY090559), ICE*Pal*Ban1 (*Providencia alcalifaciens*; accession: GQ463139), ICE*Vfl*Ind1 (*V. fluvialis*; accession: GQ463144), ICE*Pda*Spa1 (*Photobacterium damselae*; accession: AJ870986), ICE*Spu*PO1 (*Shewanella putrefaciens*; accession: CP000503), and ICE*Pmi*Chn1 (*Proteus mirabilis*; accession: KT962845).

(A) Homology of ICE*Mfu*Ind1a with other SXT/R391 ICEs

Gene name	ICE*Mfu*Ind1a gene locus id	Percentage homology of predicted proteins with SXT/R391 ICE proteins												
		ICE*Mfu*Ind1b	ICE*Pal*Ban1	ICE*Pda*Spa1	ICE*Pmi*Chn1	R391	ICE*Spu*PO1	SXT^{MO10}	ICE*Vch*Ban5	ICE*Vch*Ind4	ICE*Vch*Ind5	ICE*Vch*Mex1	ICE*Vfl*Ind1	ICE*Mpr*Chn1
xis	Ga0061065_11949	n.a	n.a	95.31	95.31	95.31	n.a	n.a	95.08	95.31	95.08	95.31	n.a	n.h
int	Ga0061065_11948	97.46[#]	97.95[#]	97.13[#]	97.95[#]	97.54[#]	98.36[#]	96.72[#]	97.95[#]	96.72[#]	97.95[#]	97.95[#]	97.95[#]	97.54[#]
int	Ga0061065_11947	96.64[#]	95.97[#]	96.64[#]	95.97[#]	95.97[#]	96.64[#]	96.64[#]	95.97[#]	96.64[#]	95.97[#]	96.64[#]	95.97[#]	96.64[#]
s002	Ga0061065_11946	95.68	89.89	88.76	89.89	91.01	87.64	89.89	84.21	89.89	89.89	96.63	89.89	88.76
s003	Ga0061065_11945	99.29	96.3	96.3	96.3	96.3	95.99	96.3	96.3	96.3	96.3	95.37	96.3	95.99
mobI	Ga0061065_11944	95.97	97.16	ab	ab	ab	98.58	ab	97.16	97.87	97.16	99.29	97.16	97.87
rumB	Ga0061065_11943	97.46	90.69[#]	ab	90.69[#]	97.63	96.21	90.69[#]	90.69[#]	90.69[#]	90.69[#]	96.68	90.69[#]	95.6[#]
rumB	Ga0061065_11943	-	97.44[#]	-	97.44[#]	-	-	98.72[#]	98.72[#]	98.72[#]	98.72[#]	-	98.72[#]	98.7[#]
rumA	Ga0061065_11942	87.25	85.23	84.56	84.56	86.58	86.58	85.91	85.91	85.91	85.91	85.91	85.91	85.91
s024	Ga0061065_11941	84.52	96.68	97.02	96.35	97.02	96.68	97.35	96.01	97.35	96.01	96.35	97.34	96.68
s025	Ga0061065_11940	ab	97.98	78	91.92	81.72	90.91	77	90.91	77	90.91	89.9	89.9	90.91
s026	Ga0061065_11939	ab	91.59	61.77	85	61.09	92.23	61.77	91.94	61.77	91.94	94.55	92.88	89.1
mfuind1a-1	Ga0061065_11938	n.h	n.h	n.h	n.h	n.h	n.h	n.h	n.h	n.h	n.h	95.75	n.h	n.h
mfuind1a-2	Ga0061065_11937	n.h	n.h	n.h	n.h	n.h	n.h	n.h	n.h	n.h	n.h	40.63	n.h	n.h
mfuind1a-4	Ga0061065_11935	n.h	n.h	n.h	n.h	n.h	n.h	n.h	n.h	n.h	n.h	97.87	n.h	n.h
traI	Ga0061065_11932	92.64[#]	93.57	91.76	94.55	92.88	92.04	92.04	93.44	92.04	93.44	92.88	n.a	92.74
traD	Ga0061065_11931	n.a	99.5	99.67	98.84	99.5	99.67	99.33	99.34	99.5	99.34	99.17	99.12	99.01
s091	Ga0061065_11930	n.a	96.77	ab	97.85	97.31	95.16	ab	96.24	94.62	96.24	95.16	n.a	97.31
traJ	Ga0061065_11929	n.a	96.21	97.63	97.63	97.16	98.1	97.16	97.16	97.16	97.16	96.21	n.a	97.16
mfuind1a-7	Ga0061065_11928	n.a	n.h	95.38	n.h	95.9	94.36	n.h	94.36	n.h	94.36	n.h	93.44	93.55
mfuind1a-8	Ga0061065_11927	n.a	n.h	94.94	n.h	96.2	94.94	n.h	n.h	n.h	n.h	n.h	n.h	n.h
traL	Ga0061065_11926	n.a	98.92	100	100	98.92	97.85	98.92	100	98.92	100	100	98.92	98.92
traE	Ga0061065_11925	99.04[#]	98.56	99.04	99.23	99.23	99.04	98.56	99.04	98.56	99.04	98.08	98.56	98.56
traK	Ga0061065_11924	91.3	92.28	91.64	92.31	93.65	92.31	93.98	93.29	93.96	93.29	93.96	91.61	91.64
traB	Ga0061065_11923	80.14	79.91	79.91	80.42	80.42	80.14	79.91	80.14	79.91	80.14	80.42	n.a	79.67
traV	Ga0061065_11922	80.53	80	78.24	78.7	78.7	78.7	78.24	81.05	78.24	78.24	81.05	80.65	78.7
traA	Ga0061065_11921	91.41	92.19	92.19	91.41	91.41	92.19	92.19	91.41	92.19	91.41	92.97	90.62	91.41
mfuind1a-9	Ga0061065_11920	92.86	n.h	n.h	n.h	96.23	n.h	n.h	n.h	n.h	n.h	96.43	n.h	n.h
mfuind1a-10	Ga0061065_11919	96.59	n.h	n.h	n.h	95.45	n.h	n.h	n.h	n.h	n.h	95.45	n.h	n.h
mfuind1a-11	Ga0061065_11918	n.h	n.h	99.12	n.h	n.h	n.h	n.h	n.h	n.h	n.h	n.h	n.h	n.h
mfuind1a-12	Ga0061065_11917	n.h	n.h	96.75	n.h	n.h	n.h	n.h	n.h	n.h	n.h	n.h	n.h	n.h

Gene name	ICE*Mfu*Ind1a gene locus id	Percentage homology of predicted proteins with SXT/R391 ICE proteins												
		ICE*Mfu*Ind1b	ICE*Pal*Ban1	ICE*Pda*Spa1	ICE*Pmi*Chn1	R391	ICE*Spu*PO1	SXT^{MO10}	ICE*Vch*Ban5	ICE*Vch*Ind4	ICE*Vch*Ind5	ICE*Vch*Mex1	ICE*Vfl*Ind1	ICE*Mpr*Chn1
mfuind1a-14	Ga0061065_11915	n.h	n.h	97.35	n.h	n.h	n.h	n.h	n.h	n.h	n.h	n.h	n.h	n.h
dsbC	Ga0061065_11914	95.22	95.22	95.22	95.65	95.65	95.65	95.65	95.22	95.65	95.22	95.2	95.65	95.65
traC	Ga0061065_11913	95.74	95.74	95.87	95.99	95.99	95.87	96.12	95.99	96.12	95.99	96.12	95.87	95.74
orf92	Ga0061065_11912	84.35	85.22	ab	ab	ab	85.22	ab	85.22	85.22	85.22	85.22	88.17	84.35
trhF	Ga0061065_11911	84.62	85.21	84.02	85.21	85.8	84.62	82.84	84.62	82.84	84.62	85.8	83.43	83.43
traW	Ga0061065_11910	89.97	91.03	89.45	90.77	90.24	89.71	90.5	n.a	90.5	n.a	n.a	91.03	91.03
traU	Ga0061065_1199	92.98	93.27	92.98	93.57	92.4	93.27	93.57	93.86	93.85	93.86	93.57	92.69	92.69
traN	Ga0061065_1198	73.02	73.02	73.02	73.27	72.94	72.86	73.35	72.53	73.02	72.53	72.94	73.02	73.11
mfuind1a-15	Ga0061065_1197	n.h	n.h	n.h	n.h	n.h	79.37	80.62	81.94	80.62	81.94	n.h	n.h	n.h
s063	Ga0061065_1196	89.73	89.19	89.19	89.73	88.11	90.22	89.19	88.11	89.19	88.11	88.65	87.03	87.03
s089	Ga0061065_1195	69.44	70.37	70.37	ab	70.37	69.44	ab	69.44	68.52	69.44	65.74	68.09	70.37
ssb	Ga0061065_1194	91.37	91.37	91.37	91.37	90.65	90.65	91.37	90.65	91.37	90.65	91.37	90.65	90.65
bet	Ga0061065_1193	94.85	94.49	94.85	94.85	94.85	94.85	94.49	94.85	94.49	94.85	94.85	94.49	94.85
exo	Ga0061065_1192	97.93	98.22	98.52	97.93	97.63	97.93	98.52	97.93	98.52	97.93	98.22	98.52	98.52
s067	Ga0061065_1191	97	98	97	97	97	97	96	97	96	97	97	98	98
s073	Ga0061065_1331	87.02	87.02	ab	75.78	87.79	87.02	89.31	87.02	89.31	87.02	87.79	87.79	75.78
mfuind1a-16	Ga0061065_1332	n.h	n.h	n.h	n.h	98.3	n.h	n.h	n.h	n.h	n.h	n.h	n.h	n.h
traF	Ga0061065_1333	93.31	95.54	94.9	92.9	94.59	93.63	94.9	95.22	94.9	95.22	97.45	93.63	93.63
traH	Ga0061065_1334	98.7	98.92	99.13	98.48	99.13	99.35	99.13	98.7	99.13	98.7	98.7	98.27	98.7
traG	Ga0061065_1335	98.61	100[#]	100[#]	100[#]	100[#]	100[#]	100[#]	100[#]	100[#]	100[#]	100[#]	100[#]	100[#]
traG	Ga0061065_11487	100	98.3[#]	98.47[#]	98.56[#]	97.54[#]	98.56[#]	98.56[#]	98.3[#]	98.56[#]	98.3[#]	98.47[#]	98.3[#]	98.98[#]
eex	Ga0061065_11486	87.41	61.54	85.31	87.41	60.56	87.41	85.31	60.84	85.31	60.84	60.14	86.71	87.41
setC	Ga0061065_11485	85.06	85.06	85.06	85.06	85.06	85.06	85.06	85.06	85.63	85.06	85.06	85.06	85.06
setD	Ga0061065_11484	90.91	89.9	90.91	90.91	90.91	89.9	90.91	89.9	90.91	89.9	88.89	89.9	90.91
s082	Ga0061065_11483	75.12	75.27	ab	75.27	75.82	75.27	75.27	75.27	75.27	75.27	n.a	75.27	75.82
s083	Ga0061065_11482	81.91	80.79	80.54	72.38	80	80.79	72.38[#]	80.79	80.79	80.79	n.a	80.3	80.3
s083	Ga0061065_11482	-	-	-	-	-	-	88.76[#]	-	-	-	-	-	-
s084	Ga0061065_11481	86.97	87.68	88.03	88.03[#]	88.38	86.97	83.93	87.68	87.32	87.68	88.03	88.03	87.68
s084	Ga0061065_11481	-	-	-	84.52[#]	-	-	-	-	-	-	-	-	-
croS/setQ	Ga0061065_11480	75	76.32	76.32	76.32	n.h	75	76.32	76.32	76.32	76.32	75	76.32	76.32
setR	Ga0061065_11479	78.14	78.14	78.14	78.14	78.14	78.14	78.14	78.14	78.14	78.14	78.14	78.14	78.14

(B) Homology of ICE*Mfu*Ind1b with other SXT/R391 ICEs

Gene name	ICE*Mfu*Ind1b gene locus id	Percentage homology of predicted proteins with SXT/R391 ICE proteins											
		ICE*Pal*Ban1	ICE*Pda*Spa1	ICE*Pmi*Chn1	R391	ICE*Spu*PO1	SXT^{MO10}	ICE*Vch*Ban5	ICE*Vch*Ind4	ICE*Vch*Ind5	ICE*Vch*Mex1	ICE*Vfl*Ind1	ICEMprChn1
int	Ga0061065_12222	99.75	99.49	99.75	99.49	99.75	99.24	99.75	99.24	99.75	100	99.75	98.98
s002	Ga0061065_12221	95.51	97.75	98.88	96.63	95.51	98.88	92.98	98.88	95.51	93.26	95.51	96.63
s003	Ga0061065_12220	99.38	98.77	99.38	99.38	98.77	98.77	99.38	98.77	99.38	98.46	99.38	99.07
mobI	Ga0061065_12219	97.87	ab	ab	ab	99.29	ab	97.87	98.58	97.87	98.58	97.87	98.58
rumB	Ga0061065_12218	91.76[#]	ab	91.76[#]	97.01	96.92	91.49[#]	91.76[#]	91.49[#]	91.76[#]	96.81	91.76[#]	97.07[#]
rumB	Ga0061065_12218	97.44[#]	-	95.6[#]	-	-	95.79[#]	98.72[#]	98.72[#]	98.72[#]	-	98.72[#]	98.7[#]
rumA	Ga0061065_12217	97.99	97.32	97.32	98.66	99.33	98.66	98.66	98.66	98.66	98.66	98.66	98.66
s024	Ga0061065_12216	84.52	85.71	83.33	85.71	84.52	84.52	84.52	84.52	84.52	85.71	84.52	85.71
Mfuind1b-11	Ga0061065_1225	n.h	n.h	n.h	n.h	n.h	n.h	n.h	n.h	n.h	n.h	n.h	61.46
Mfuind1b-11	Ga0061065_1224	n.h	n.h	n.h	n.h	n.h	n.h	n.h	n.h	n.h	n.h	n.h	97.85
traI	Ga0061065_1221	93.92	92	93.44	92.96	93.44	91.68	92.8	91.68	92.8	92.64	n.a	92
traE	Ga0061065_1231	98.56	99.04	99.23	99.23	99.04	98.56	99.04	98.56	99.04	98.08	98.56	98.56
traK	Ga0061065_1232	95.97	96.32	95.32	95.65	96.32	95.32	95.3	95.3	95.3	94.63	96.64	96.66
traB	Ga0061065_1233	99.07	99.77	98.37	99.53	98.14	98.83	99.53	98.83	99.53	98.6	n.a	99.53
traV	Ga0061065_1234	96.84	96.84	97.37	97.89	97.37	96.84	97.89	96.84	97.89	97.89	96.24	96.32
traA	Ga0061065_1235	99.22	99.22	98.44	98.44	97.66	99.22	96.88	99.22	96.88	98.44	97.66	98.44
mfuind1b-15	Ga0061065_1236	n.h	n.h	n.h	96.23	n.h	n.h	n.h	n.h	n.h	92.86	n.h	n.h
mfuind1b-16	Ga0061065_1237	n.h	n.h	n.h	98.86	n.h	n.h	n.h	n.h	n.h	98.86	n.h	n.h
dsbC	Ga0061065_1238	99.13	99.13	99.57	99.57	99.57	99.57	99.13	99.57	99.13	99.13	99.57	99.57
traC	Ga0061065_1239	99	99.12	99.25	99.25	99.12	99.25	99.25	99.25	99.25	99.37	99.12	99
orf92	Ga0061065_12310	97.39	ab	ab	ab	98.26	ab	97.39	97.39	97.39	97.39	97.85	96.52
trhF	Ga0061065_12311	98.82	97.06	98.24	98.77	97.06	98.24	98.82	98.24	98.82	97.65	96.47	96.47
traW	Ga0061065_12312	95.99	95.72	97.06	96.52	94.92	96.26	n.a	96.26	n.a	n.a	95.72	95.72
traU	Ga0061065_12313	98.54	98.83	98.83	98.25	98.54	98.83	98.83	99.08	98.83	99.42	99.12	99.12
traN	Ga0061065_12314	98.13	98.29	98.13	97.89	97.89	97.91	97.56	97.8	97.56	96.67	98.46	98.54
s063	Ga0061065_12321	98	98.5	98	98	97.5	98.5	97.5	98.5	97.5	98.5	97	97
s089	Ga0061065_12322	99.07	95.37	ab	98.15	99.07	ab	92.59	97.22	92.59	93.52	98.94	99.07
ssb	Ga0061065_12323	97.84	100	100	97.84	99.28	96.4	97.12	96.4	97.12	98.56	99.28	99.28
bet	Ga0061065_12324	99.63	100	100	100	99.63	99.63	100	99.63	100	100	99.63	100
orfZ	Ga0061065_12325	97.92	-	-	-	-	-	96.53	99.31	96.53	98.61	97.92	93.62
exo	Ga0061065_12326	98.52	98.82	98.82	98.52	98.82	98.82	98.82	98.82	98.82	98.52	98.82	98.82
s067	Ga0061065_12327	99.69	99.37	99.69	99.06	99.37	99.69	99.37	99.69	99.37	99.69	99.37	99.69
s088	Ga0061065_12328	96.47	96.08	99.22	96.86	96.86	89.31	96.47	97.24	96.47	96.47	97.25	97.25
s070	Ga0061065_13011	97.56	96.34	97.56	100	100	98.78	98.78	98.78	98.78	97.56	97.56	97.56
s071	Ga0061065_13010	96.36	97.58	97.58	97.58	96.36	98.18	96.36	98.18	96.36	98.18	97.58	97.58
s092	Ga0061065_1309	95.58	ab	94.69	92.92	97.35	ab	94.69	97.35	94.69	96.46	94.69	94.69
s072	Ga0061065_1308	96.64	95.8	98.6	97.2	95.24	97.55	96.64	96.92	96.64	96.64	98.32	98.6
s073	Ga0061065_1307	89.79	ab	84.91	89.36	89.79	91.49	90.21	91.49	90.21	90.21	90.17	84.91
traF	Ga0061065_1303	95.54	95.22	93.87	97.77	96.82	95.86	95.22	95.86	95.22	93.31	96.5	94.59
traH	Ga0061065_1302	98.92	98.7	98.27	98.7	98.92	99.13	98.7	99.13	98.7	98.7	97.84	98.48
traG	Ga0061065_1301	100[#]	100[#]	100[#]	100[#]	100[#]	100[#]	100[#]	100[#]	100[#]	100[#]	100[#]	100[#]
traG	Ga0061065_109135	98.89[#]	99.17[#]	99.03[#]	97.5[#]	98.89[#]	98.89[#]	98.89[#]	98.89[#]	98.89[#]	99.03[#]	98.75[#]	98.89[#]
eex	Ga0061065_109134	70.63	95.8	97.9	69.72	96.5	96.5	69.93	96.5	69.93	69.23	97.2	96.5
setC	Ga0061065_109133	99.44	100	99.44	99.44	100	98.87	99.44	99.44	99.44	100	99.44	100

Gene name	ICE*Mfu*Ind1b gene locus id	Percentage homology of predicted proteins with SXT/R391 ICE proteins											
		ICE*Pal*Ban1	ICE*Pda*Spa1	ICE*Pmi*Chn1	R391	ICE*Spu*PO1	SXT^{MO10}	ICE*Vch*Ban5	ICE*Vch*Ind4	ICE*Vch*Ind5	ICE*Vch*Mex1	ICE*Vfl*Ind1	ICEMprChn1
s082	Ga0061065_109131	100	ab	98.35	97.8	100	100	100	100	100	n.a	99.45	98.9
s083	Ga0061065_109130	99.09	98.51	97.54	98.02	99.09	97.54	99.09	99.09	99.09	n.a	98.18	98.64
s083	Ga0061065_109130	-	-	-	-	-	97.78[#]	-	-	-	-	-	-
s084	Ga0061065_109129	98.62	98.27	98.62[#]	98.27	99.65	99.42[#]	98.62	98.96	98.62	98.62	98.62	96.89
s084	Ga0061065_109129	-	-	100[#]	-	-	-	-	-	-	-		-
croS/setQ	Ga0061065_109128	97.59	97.59	96.39	n.h	100	97.59	97.59	97.59	97.59	96.39	97.59	96.39
setR	Ga0061065_109127	100	100	100	100	100	100	100	100	100	100	100	100
prfC	Ga0061065_109126	n.a	n.a	84.12	n.a	78.64	n.a	n.a	n.a	n.a	96.2	89.04	69.19

n.h, no homology based on BLASTP search with e-value cut-off of 1e-5, query coverage > 90 %, and identity > 40 %.

n.a, sequence not available.

[#], partial protein sequences.

ab, absent or deleted.

Supplementary Table S3. Descriptions of genes or ORFs present in the hotspot regions of ICE*Mfu*Ind1a.

Hotspot region	ICE*Mfu*Ind1a gene/ORF id	Gene name	Length (bp)	Hotspot gene product name	Accession no. of protein homolog in ICEberg database	% similarity with homolog
HS-5 (*s026-traI*)	Ga0061065_11938	mfuind1a-1	1560	Type-I restriction enzyme M subunit	*Vibrio cholerae* Mex1; ACV96414.1	96
	Ga0061065_11937	mfuind1a-2	1245	Type-I restriction enzyme S subunit	*Vibrio cholerae* Mex1; ACV96472.1	41
	Ga0061065_11936	mfuind1a-3	1176	-	-	n.h
	Ga0061065_11935	mfuind1a-4	3117	Type-I restriction enzyme R subunit	*Vibrio cholerae* Mex1; ACV96447.1	98
	Ga0061065_11934	mfuind1a-5	564	-	-	n.h
	Ga0061065_11933	mfuind1a-6	912	-	-	n.h
HS-1 (*traJ-traL*)	Ga0061065_11928	mfuind1a-7	588	Hypothetical protein	*Providencia rettgeri*; AAM08045.1	96
	Ga0061065_11927	mfuind1a-8	240	Hypothetical protein	*Providencia rettgeri*; AAM08045.1	96
HS-2 (*traA-s054*)	Ga0061065_11920	mfuind1a-9	507	Acetyltransferase (GNAT) family	*Vibrio cholerae* Mex1; ACV96420.1	96
	Ga0061065_11919	mfuind1a-10	267	Conserved hypothetical protein	*Vibrio cholerae* Mex1; ACV96425.1	95
	Ga0061065_11918	mfuind1a-11	2730	Hypothetical protein	*Vibrio cholerae* MJ-1236; ACQ61857.1	99
	Ga0061065_11917	mfuind1a-12	1110	Hypothetical protein	*Vibrio cholerae* MJ-1236; ACQ61856.1	98
	Ga0061065_11916	mfuind1a-13	1533	Hypothetical protein	*Vibrio cholerae* MJ-1236; ACQ61855.1	99
	Ga0061065_11915	mfuind1a-14	1446	Type 4 fimbriae expression regulatory protein pilR	*Vibrio cholerae* MJ-1236; ACQ61854.1	99
HS-4 (*s073-traF*)	Ga0061065_1197	mfuind1a-15	693	Endonuclease-1	*Vibrio cholerae* Ind5; ACV96406.1	82
HS-3 (*traN-s063*)	Ga0061065_1332	mfuind1a-16	1065	Hypothetical protein	*Providencia* rettgeri; AAM08049.1	98

n.h, No homology

Supplementary Table S4. Descriptions of genes or ORFs present in the hotspot regions of ICE*Mfu*Ind1b.

Hotspot region	ICE*Mfu*Ind1b gene/ORF id	Gene name	Length (bp)	Hotspot gene product name	Accession no. of protein homolog in ICEberg database	% similarity with homolog
HS-5 (*s024-traI*)	Ga0061065_12215	mfuind1b-1	1038	Transcriptional regulator, AraC family	*Polaromonas naphthalenivorans* CJ2; ABM39468.1	25
	Ga0061065_12214	mfuind1b-2	1485	Putative gamma-glutamyl-gamma-aminobutyraldehyde dehydrogenase	*Klebsiella pneumoniae* 342; ACI07089.1	27
	Ga0061065_12213	mfuind1b-3	1779	-	-	n.h
	Ga0061065_12212	mfuind1b-4	900	DNA-binding transcriptional regulator, LysR	*Bordetella petrii*; CAP41590.1	35
	Ga0061065_12211	mfuind1b-5	1512	Histidine ammonia lyase	*Klebsiella pneumoniae* 342; ACI08660.1	32
	Ga0061065_12210	mfuind1b-6	2013	-	-	n.h
	Ga0061065_1229	mfuind1b-7	1272	-	-	n.h
	Ga0061065_1228	mfuind1b-8	1050	-	-	n.h
	Ga0061065_1227	mfuind1b-9	240	-	-	n.h
	Ga0061065_1226	mfuind1b-10	1254	-	-	n.h
	Ga0061065_1225	mfuind1b-11	885	Type-III restriction system methylase	*Bordetella petrii*; CAP44585.1	37
	Ga0061065_1224	mfuind1b-12	3078	Type-III restriction enzyme	*Bordetella petrii*; CAP44586.1	36
	Ga0061065_1223	mfuind1b-13	282	-	-	n.h
	Ga0061065_1222	mfuind1b-14	474	-	-	n.h
HS-1 (*traJ-traL*)	Sequence not known	-	-	-	-	-
HS-2 (*traA-s054*)	Ga0061065_1236	mfuind1b-15	507	Acetyltransferase (GNAT) family	*Vibrio cholerae* Mex1; ACV96420.1	93
	Ga0061065_1237	mfuind1b-16	267	Conserved hypothetical protein	*Vibrio cholerae* Mex1; ACV96425.1	99
HS-4 (*s073-traF*)	Ga0061065_12315	mfuind1b-17	684	-	-	n.h
	Ga0061065_12316	mfuind1b-18	897	-	-	n.h
	Ga0061065_12317	mfuind1b-19	1131	-	-	n.h
	Ga0061065_12318	mfuind1b-20	531	-	-	n.h
	Ga0061065_12319	mfuind1b-21	1317	Conserved hypothetical protein	*Bordetella petrii*; CAP41687.1|	22
	Ga0061065_12320	mfuind1b-22	324	-	-	n.h
HS-3 (*traN-s063*)	Ga0061065_1306	mfuind1b-23	1782	Signal transduction GGDEF/EAL domain protein	*Burkholderia multivorans* ATCC 17616; BAG42888.1	33
	Ga0061065_1305	mfuind1b-24	2400	Probable signaling protein	*Bordetella petrii*; CAP41698.1	31
	Ga0061065_1304	mfuind1b-25	1557	Hypothetical protein	*Bordetella petrii*; CAP41755.1	37

n.h, No homology

Supplementary Table S5. Descriptions of genes or ORFs present in the hotspot regions of ICE*Mpr*Chn1.

Hotspot region	ICE*Mpr*Chn1 gene/ORF id	Gene name	Length (bp)	Hotspot gene product name	Accession no. of protein homolog in ICEberg database	% similarity with homolog
HS-5 (*s026-traI*)	D104_09935	mprchn1-1	3333	DNA-dependent ATPase, SNF2 family protein	*Bordetella petrii*; CAP44583.1	41
	D104_09940	mprchn1-2	687	Hypothetical protein	*Bordetella petrii*; CAP44584.1	26
	D104_09945	mprchn1-3	1947	Type-III restriction system methylase	*Bordetella petrii*; CAP44585.1	36
	D104_09950	mprchn1-4	3078	Type III restriction enzyme R subunit	*Bordetella petrii*; CAP44586.1	36
	D104_09955	mprchn1-5	855	-	-	n.h
HS-1 (*traJ-traL*)	D104_09980	mprchn1-6	1557	Transposase	*Vibrio fluvialis* Ind1; ACV96587.1	99
	D104_09985	mprchn1-7	96	-	-	n.h
	D104_09995	mprchn1-8	219	-	-	n.h
	D104_10000	mprchn1-9	243	-	-	n.h
	D104_10005	mprchn1-10	708	-	-	n.h
	D104_10010	mprchn1-11	372	-	-	n.h
	D104_10015	mprchn1-12	693	TetR family transcriptional regulator	*Burkholderia xenovorans* LB400; ABE31807.1	30
	D104_10020	mprchn1-13	1047	Hypothetical protein	*Bacteroides ovatus*; CAD89793.1	28
	D104_10025	mprchn1-14	3042	Transporter, hydrophobe/amphiphile efflux-1 (HAE1) family	*Shewanella* sp. W3-18-1; ABM23977.1	25
	D104_10030	mprchn1-15	615	-	-	n.h
	D104_10035	mprchn1-16	234	-	-	n.h
	D104_10040	mprchn1-17	1305	Putative transposase	*Streptococcus suis* SC84; CAZ51643.1	22
	D104_10445	mprchn1-18	1173	Conserved hypothetical protein	*Vibrio fluvialis* Ind1; ACV96594.1	99
	D104_10450	mprchn1-19	696	Transposition helper protein	*Vibrio fluvialis* Ind1; ACV96542.1	100
	D104_10455	mprchn1-20	573	Conserved hypothetical protein	*Vibrio fluvialis* Ind1; ACV96517.1	100
HS-2 (*traA-s054*)	D104_10490	mprchn1-21	834	Conserved hypothetical protein	*Vibrio fluvialis* Ind1; ACV96573.1	100
	D104_10495	mprchn1-22	702	Hypothetical protein	*Vibrio cholera*; AAL59716.1	99
	D104_10500	mprchn1-23	408	MerR family transcriptional regulator	*Burkholderia xenovorans LB400*; ABE31843.1	99
	D104_10505	mprchn1-24	885	Heavy metal efflux pump, CDF family	*Burkholderia xenovorans LB400*; ABE31842.1	98
HS-4 (*s073-traF*)	D104_04195	mprchn1-25	434	Hypothetical protein	*Vibrio cholerae* MJ-1236; ACQ61846.1	99
	D104_04190	mprchn1-26	663	Hypothetical protein	*Proteus mirabilis*; BAI48432.1	100

n.h, No homology
